# Multivariate and Phylogenetic Analyses Assessing the Response of Bacterial Mat Communities from an Ancient Oligotrophic Aquatic Ecosystem to Different Scenarios of Long-Term Environmental Disturbance

**DOI:** 10.1371/journal.pone.0119741

**Published:** 2015-03-17

**Authors:** Silvia Pajares, Valeria Souza, Luis E. Eguiarte

**Affiliations:** Departamento de Ecología Evolutiva, Instituto de Ecología, Universidad Nacional Autónoma de México, CU, Mexico City, Mexico; CNRS - Université Lyon 1, FRANCE

## Abstract

Understanding the response of bacterial communities to environmental change is extremely important in predicting the effect of biogeochemical modifications in ecosystem functioning. The Cuatro Cienegas Basin is an ancient oasis in the Mexican Chihuahuan desert that hosts a wide diversity of microbial mats and stromatolites that have survived in extremely oligotrophic pools with nearly constant conditions. However, thus far, the response of these unique microbial communities to long-term environmental disturbances remains unexplored. We therefore studied the compositional stability of these bacterial mat communities by using a replicated (3x) mesocosm experiment: a) Control; b) Fluct: fluctuating temperature; c) 40C: increase to 40 ºC; d) UVplus: artificial increase in UV radiation; and f) UVmin: UV radiation protection. In order to observe the changes in biodiversity, we obtained 16S rRNA gene clone libraries from microbial mats at the end of the experiment (eight months) and analyzed them using multivariate and phylogenetic tools. Sequences were assigned to 13 major lineages, among which Cyanobacteria (38.8%) and Alphaproteobacteria (25.5%) were the most abundant. The less extreme treatments (Control and UVmin) had a more similar composition and distribution of the phylogenetic groups with the natural pools than the most extreme treatments (Fluct, 40C, and UVplus), which showed drastic changes in the community composition and structure, indicating a different community response to each environmental disturbance. An increase in bacterial diversity was found in the UVmin treatment, suggesting that protected environments promote the establishment of complex bacterial communities, while stressful environments reduce diversity and increase the dominance of a few Cyanobacterial OTUs (mainly *Leptolyngbya sp*) through environmental filtering. Mesocosm experiments using complex bacterial communities, along with multivariate and phylogenetic analyses of molecular data, can assist in addressing questions about bacterial responses to long-term environmental stress.

## Introduction

It is predicted that global environmental change will severely affect aquatic ecosystems due to the projected alteration of UV irradiance and temperature regimes [1–3]. It is also expected that bacterial communities will play a key role in ecosystem response to such environmental change due to their central role in nutrient cycling [4]. To accurately forecast how biogeochemical processes will respond to global environmental change, we must consider microbial community composition and/or adaptation of these communities to resource environments [5,6]. Thus, a better understanding of which mechanisms drive microbial community assembly and how these will be affected by environmental change is therefore of vital importance in the prediction of the fate of aquatic ecosystems.

In recent years, there have been significant advances in the development of phylogenetic frameworks that measure the relative importance of processes shaping microbial community structure [7–10]. Studying the phylogenetic structure of communities can provide valuable insights into the evolutionary and ecological processes that drive community assembly as well as the response of communities to environmental changes [11,12]. These techniques have been further enriched by multivariate statistical analyses, which can be applied to address fundamental and complex questions in microbial ecology [13], such as whether microbial diversity responds to the same factors as macroorganism diversity [14,15]. Thus, the full potential of 16S rRNA gene analyses in combination with multivariate statistics provides the appropriate tools for describing variations in bacterial community composition while linking such variations to changes in the environment.

Microbial mats are self-sustaining laminated organo-sedimentary ecosystems composed of tightly interacting microorganisms. This multilayered ecosystem generates a diverse array of micro-niches in which different functional guilds can thrive; meanwhile, the spatial closeness allows for the development of complex metabolite exchange networks that ensure survival even under extreme conditions [16]. Microbial mats have a strong historical significance in terms of their role in the ecology of early Earth [17] and their capabilities for biochemical transformations and energy flows [18]. However, there have been very few molecular surveys focusing on the response of such communities to environmental disturbances [19,20], given the inherent difficulty of long-term studies. On the other hand, the geographical distribution of modern microbial mats is currently restricted to only a few aquatic systems, one of which is the Cuatro Cienegas Basin (CCB), an oasis in the Chihuahuan desert of Mexico. These habitats provide windows of understanding into how life persisted in the past and how microbial ecosystems will respond to future global change [21].

The CCB is an ancient ecosystem composed of several pools dominated by microbial mats and stromatolites that have evolved in relative isolation and under nutrient constraints [22,23]. This ecosystem faces extreme oligotrophy, which is severely limited by phosphorous (the N-to-P ratio of total nutrients in this system is extremely high: >100:1 [22,24]). Nevertheless, the aquatic microbiota have a high level of diversity and endemism [23,25], complex metabolisms [18,26], and long-term population stability [21]. These communities inhabit slightly thermal springs (CA. 30°C) with a constant environment due to the continuous flow of deep hot water to the springs [27]; they form the basis of food webs in this unique setting [28]. Thus, the microbial mats of CCB are suitable model systems that can be used to evaluate how ecosystems respond to “press environmental disturbances” (continuous disturbances that may arise sharply but reach a constant level that is maintained over a long period [29,30]). These disturbances include increased temperature and/or UV radiation, which cannot be tested as easily as with other ecosystems [19].

To study how these microbial ecosystems respond to long-term environmental changes, we can construct replicated experimental systems that enable us to recreate, in a simpler way, the natural habitat of the microbial mats while simultaneously allowing manipulation [31]. Such simplicity enables a high degree of experimental control and replicability to address many questions in the study of microbial community assembly that are difficult to reach through other kind of experiments or field observations [32].

In a previous eight-month experiment [33], we reported drastic changes over time in an oligotrophic bacterioplankton community from CCB to different types of continuous environmental stress. However, studies at CCB observed that the bacterial genotypes, even within a genus, in the water column differ from the ones in the sediment [34]. Hence, in order to assess this ecosystem’s bacterial community susceptibility to “press environmental disturbances”, we need to measure the influence of such changes in both bacterioplankton and bacterial mats diversity. Herein, we investigated if the bacterial mat community composition and structure from the same mesocosm experiment responded to these long-term perturbations in a similar way as the bacterioplankton community.

## Material and Methods

### Ethics statement

All necessary permits were obtained for this study. SEMARNAT (The Ministry of Environment and Natural Resources for Mexico) provided the research permits SGPA/DGVS/08768/08 and SGPA/DGVS/07693/09 to conduct this study in the CCB protected area.

### Experimental design

**Fig 1:**
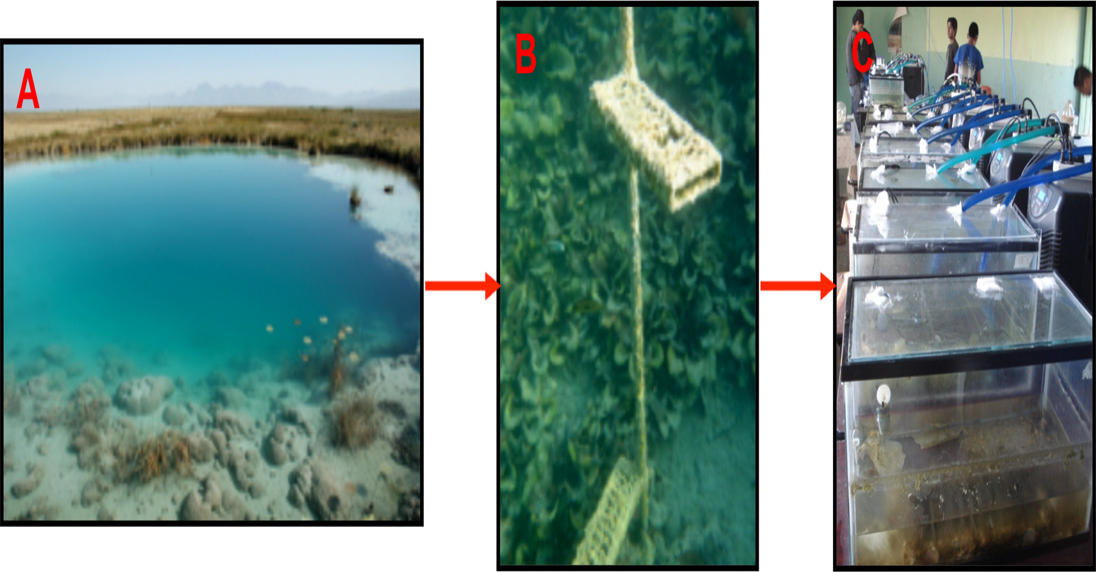
Mesocosm experiment with “synthetic” microbial mats from CCB. A: Poza Azul; B: “microbial mat catchers” (trays with frosted glass slides) in the natural pools; C: mesocosm experiment.

The experimental design was implemented as described in Pajares et al. [33]. Briefly, in July 2009, we constructed 15 mesocosms in 40 L sterilized tanks with 600 L of composite water samples from three small adjacent natural pools in CCB (Coahuila State, Mexico: 26° 49.4` N, 102° 00.9` W). In order to replicate these complex microbial mat communities without perturbing their natural environment, we placed acrylic trays with frosted glass slides in the pools to seed microbial mat-forming communities (“microbial mat catchers”) two years prior to the mesocosm experiment (Fig. 1). These pools are oligotrophic springs with similar physico-chemical properties (neutral pH, oxygen-saturated, low nitrogen concentration and rich in calcium carbonates [31]); they sustain the growth of microbial mat communities at a nearly constant temperature all year long (CA. 30°C) due to a deep hydrothermal water source [27].

The mesocosm experiment consisted of five treatments, each replicated three times: a) Control: similar constant temperature (30°C) and UV intensity to the original pools; b) Fluct: fluctuating temperature (26–43°C in summer and 0–27°C in winter) and similar UV intensity to the pools; c) 40C: increased constant temperature of 40°C and similar UV intensity to the pools; d) UVplus: artificial increase in UV radiation for 12 h per day and similar temperature to the pools; and f) UVmin: reduction in UV light with acrylic filters and similar temperature to the pools. The water temperature and aerated conditions were maintained with a BOYU Chiller. In the Fluct treatment, no chiller was added, and the temperature fluctuated with the ambient temperature. A HOBO Pendant Temperature/Light Data Logger was placed into each tank to monitor variations in temperature and light. For the UVplus treatment, a UV light lamp (BioPro underwater light) was added to the tanks. The tanks were refilled with purified sterile autoclaved water when their water volume decreased by evaporation and their walls were cleaned three times a week with 96% alcohol to prevent external contamination. To replicate the natural conditions of this nutrients-limited ecosystem [22,24], no nutrients were supplied in this experiment. The mesocosms were placed at random in a well-illuminated and aerated room, 17 km away from the collection sites, at the Centro Bachillerato Tecnológico Agropecuario (CBTA22) in the village of Cuatro Cienegas.

### Environmental 16S rRNA gene clone libraries

In order to have a fair comparison, we used the same molecular technique as the one reported previously for the bacterioplankton community from the same mesocosm experiment [33]. The number of “microbial mat catchers” seeded in the natural pools two years previous to the mesocosm experiment were unfortunately not enough to sample at the beginning of the experiment. Besides, bacterial communities from both the natural pools and mesocosms change with the seasons following the natural cycle of sun irradiation and environmental fluctuations; therefore, the bacterial mat composition was not necessary to analyze at the beginning of the experiment. Thus, we obtained 16S rRNA gene clone libraries from the microbial mats developed in the glass slides seeded in each mesocosm, along with the “microbial mat catchers” from each of the three natural pools at the end of the experiment (after eight months).

Genomic DNA was extracted from the microbial mat samples with the PowerBiofilm DNA Isolation kit (Mo-Bio, USA). Three different PCR reactions per sample for amplification of 16S rRNA genes contained in 50 μl reaction volume: 20 ng of DNA, 1 x High Fidelity PCR buffer, 1.5 mM MgSO_4_, 0.2 mM of each deoxynucleotide, 500 mM of 16S rRNA universal primers set 27F/1492R [35], 1mg ml^-1^ BSA, 5% DMSO and 1U of High Fidelity Platinum Taq DNA polymerase (Invitrogen, USA). Amplification was performed as follows: initial denaturation at 94°C for 3 min, 30 cycles of denaturation at 94°C for 1 min, annealing at 52°C for 1 min and extension at 72°C for 1.2 min, followed by a final extension of 15 min at 72°C. Amplified products were purified with a QIAquick PCR purification kit (Qiagen Inc., the Netherlands) and cloned with a Topo Cloning kit (Invitrogen, USA), following the manufacturer’s instructions. Cloned inserts were isolated for sequencing using the Plasmid Miniprep kit (Millipore, USA). Partial 16S rRNA gene inserts from plasmids were sequenced with the 27F primer at the DNA Laboratory of the University of Washington (Seattle, USA).

### Phylogenetic, diversity and multivariate analyses

For comparative purposes, we followed a similar methodology to Pajares et al. [33] for the phylogenetic and diversity analyses. Briefly, we aligned the 16S rRNA sequences with an average length of 760 bp using the nearest alignment space termination (NAST) algorithm hosted at Greengenes reference database [36]. We manually corrected aligned sequences with the Bioedit program and detected and removed chimeras using the Chimera check Bellerophon program v3 [37]. We used the naïve Bayesian Classifier tool at the Ribosomal Database Project (RDP) to determine the taxonomic affiliation of the chimera-free sequences [38]. Sequences were deposited in GenBank with the following accession numbers: KJ611247—KJ611846.

Sequences were clustered into operational taxonomic units (OTUs) at 3% distance using the average neighbor method with Mothur software [39]. A neighbor-joining phylogenetic tree of representative sequences was constructed using the Quicktree program with 1,000 bootstrap replicates. The resulting phylogenetic tree was edited with the interactive Tree of Life online program [40]. In order to explore the potential habitat affinities of these clone libraries, OTUs were compared with their closest relatives (i.e., the first ten hits) with the Seqmatch tool in the RDP. Alpha diversity indices (Shannon, Simpson, and Berger-Parker), Good’s coverage estimates, richness estimates (Chao1) and rarefaction curves based on the rank-abundance of OTUs were calculated for each treatment and the pools with Mothur. Pooled data per treatment served as the experimental unit for diversity indices; due to the variable number of clones sequenced from each mesocosm, the pooled data provided a better estimate of the overall diversity in each treatment [41].

To visualize the differences in bacterial mat community structure between the pools and the mesocosms, we calculated Canberra dissimilarity distances based on the OTUs abundance in each sample; we then clustered similar communities using the Ward’s hierarchical clustering algorithm, which tries to minimize variances in agglomeration [42]. We also performed a non-metric multidimensional scaling (NMDS) analysis to depict community structure patterns in two dimensions. For this purpose, we constructed a dissimilarity matrix using the Bray-Curtis coefficient after a square-root transformation of the data to lend more weight to the rare OTUs. To test the hypothesis that environments (treatments and pools) structure the distribution of bacterial communities, permutational multivariate analysis of variance (PERMANOVA) was used with 999 permutations (p = 0.05) [43]. We carried out a multivariate homogeneity of group dispersion analysis (beta diversity) to assess the homogeneity of bacterial communities within a group of samples [44]. To analyze the distribution of bacterial orders in the pools and treatments, we constructed a heatmap of the relative abundance data using the Bray-Curtis dissimilarity distances and Ward’s hierarchical clustering algorithm. We also examined the bacterial community’s overlap between the experimental treatments using Venn diagrams. All of these analyses were performed with vegan [45] and gplots [46] packages in the R software [47].

We calculated the phylogenetic community structure using the mean pairwise distance (MPD) and mean nearest taxon distance (MNTD) of all the OTU pairs occurring in a community based on the observed community dataset [11]. The MPD measures overall clustering across the phylogeny as the average distance between all pairs of taxa in a community. The MNDT measures the extent of terminal clustering on the phylogeny by determining the minimal distance or branch length between taxa in a particular community. We calculated the differences in the phylogenetic distances between the observed and randomly generated null communities, and they were standardized using the standardized deviation of phylogenetic distances in 10,000 null communities based on the independent swap algorithm, which shuffles the taxa/sample table [48]. Samples were considered significantly overdispersed or clustered (α <0.05) if they fell above or below 95% of the randomized communities’ values, respectively. These analyses were implemented with the Picante package in the R environment [49].

## Results

We sought to assess the long-term effects of different environmental disturbances on the stability of bacterial mat communities from the oligotrophic ecosystem of the CCB. To do so, we compared the bacterial composition and phylogenetic structure of the “microbial mat catchers” from the natural pools and the different treatments in an eight-month-long mesocosm experiment.

### Bacterial community composition and habitat affiliation

**Fig 2:**
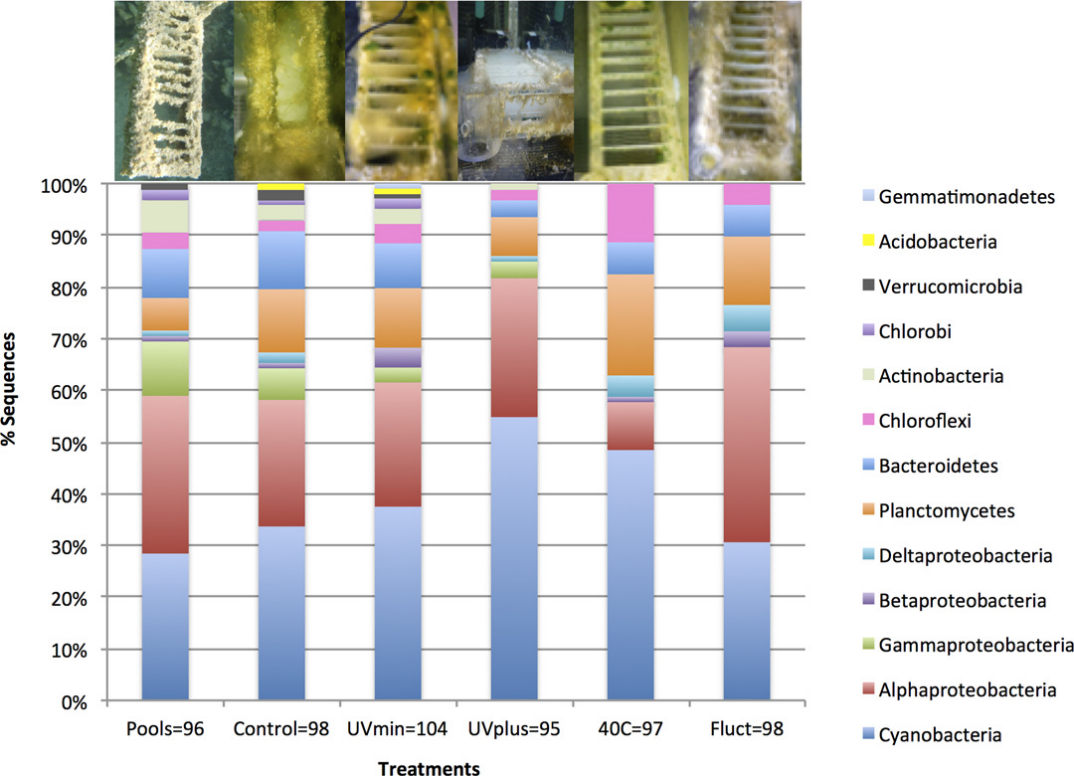
Relative abundance of bacterial taxonomic groups from the clone libraries data of the microbial mats in the treatments and the pools at the end of the experiment. The number of clones obtained from each environment is indicated below in its respective bar. The photo above each bar represents the “microbial mat catchers” in each environment.

At the end of this experiment, we recovered a total of 250 OTUs out of 600 sequences from the “microbial mat catchers”, including both the natural pools and the mesocosm experiment (S1 Fig., S1 Table). Four OTUs from 15 sequences belonged to algal chloroplasts and were excluded from the bacterial analyses. Sequences were assigned to 13 major lineages, the most abundant of which were the following: Cyanobacteria (38.8%), largely dominated by the genus *Leptolyngbya*; Alphaproteobacteria (25.5%), with several OTUs from the Rhizobiales and Rhodobacterales orders; and Planctomycetes (11.8%), mainly belonging to the Phycisphaeraceae and Planctomycetaceae families (Fig. 2, S1 Table). These findings are consistent with other studies of bacterial mat communities from CCB using 16S rRNA clone libraries and metagenomics [25,50]. Conversely, these microbial mats showed a different composition from the bacterioplankton of the same pools, which are dominated by Actinobacteria belonging to the Microbacteriaceae family [31]. In this study, a large proportion of OTUs resembled organisms associated with freshwater environments (44%; Table 1 and S2 Fig.), from where a majority also had a close affinity with bacterioplankton from this mesocosm experiment, especially Cyanobacteria belonging to the Pseudanabaenaceae and Noctocaceae families, as well as Alphaproteobacteria belonging to the Rhizobiales and Rhodobacterales orders [33]. The next largest habitat affiliation was microbial mats ecosystems (15%), mainly for bacterial OTUs belonging to Sphingobacteriales (phylum Bacteroidetes) from CCB [50] and Anaerolineaceae (phylum Chloroflexi) from Guerrero Negro [50,51]. Other important habitat affiliations included soil (9%) and sewage sludge (9%).

**Table 1:** Taxonomic assignment of the most abundant bacterial OTUs (at 97% cut-off) from the microbial mats at the end of the experiment using the Ribosomal Database Project tool for classification.

Most abundant OTUs (97%)	Putative phylum	S_ab score	Habitat	% total OTUs
*Leptolyngbya sp* (4,11,1,32,0,3)	Cyanobacteria	1	Freshwater (M)	8.5
*Leptolyngbya sp* (0,2,0,0,16,0)	Cyanobacteria	0.982	Freshwater (M)	3
Nostocaceae bacterium (3,1,8,2,0,3)	Cyanobacteria	0.973	Freshwater (M)	2.8
Pseudanabaenaceae bacterium (0,3,9,2,0,0)	Cyanobacteria	1	Freshwater (M)	2.3
Phycisphaeraceae bacterium (1,0,0,2,11,0)	Planctomycetes	0.92	Freshwater (M)	2.3
*Stenotrophomonas sp* (7,3,1,2,0,0)	Gammaproteobacteria	1	Hot Springs (Y)	2.2
*Rhodobacter sp* (2,3,2,0,1,3)	Alphaproteobacteria	0.941	Sewage sludge	1.8
*Ohtaekwangia sp* (0,2,6,1,1,0)	Bacteroidetes	0.99	Freshwater (M)	1.7
Chroococcales bacterium (0,0,0,0,0,10)	Cyanobacteria	0.945	Hot Springs (Y)	1.7
Pseudanabaenaceae bacterium (1,0,0,0,9,0)	Cyanobacteria	0.979	Freshwater (M)	1.7
Oscillatoriales bacterium (2,0,0,3,0,5)	Cyanobacteria	0.88	Lake	1.7
Brucellaceae bacterium (5,0,1,1,0,0)	Alphaproteobacteria	1	Wetland	1.2
Hyphomicrobium sp (0,1,0,0,0,6)	Alphaproteobacteria	1	Freshwater (M)	1.2
*Porphyrobacter sp* (0,3,0,3,0,1)	Alphaproteobacteria	0.906	Biofilm	1.2
Chroococcales bacterium (0,0,4,2,0,1)	Cyanobacteria	0.944	Microbial mat	1.2
Planctomycetaceae bacterium (0,0,0,0,0,7)	Planctomycetes	0.854	Groundwater	1.2
Rhodobacteraceae bacterium (0,0,4,2,0,0)	Alphaproteobacteria	1	Freshwater (M)	1
*Leptolyngbya sp* (0,0,0,0,6,0)	Cyanobacteria	0.98	Hot Springs	1
Planctomycetales bacterium (0,1,3,2,0,0)	Planctomycetes	0.876	Sediment	1

OTU designations are followed (in parenthesis) by the number of sequences represented by that OTU in each environment. These designations are presented in the following order: Pools, Control, UVmin, UVplus, 40C, and Fluct. The S_ab score represents the percentage of shared 7-mers between two sequences, which does not require alignment in the calculation. Abbreviations: M is the mesocosm experiment; Y is Yellowstone (USA). S1 Table provides the list of all OTUs.

**Fig 3:**
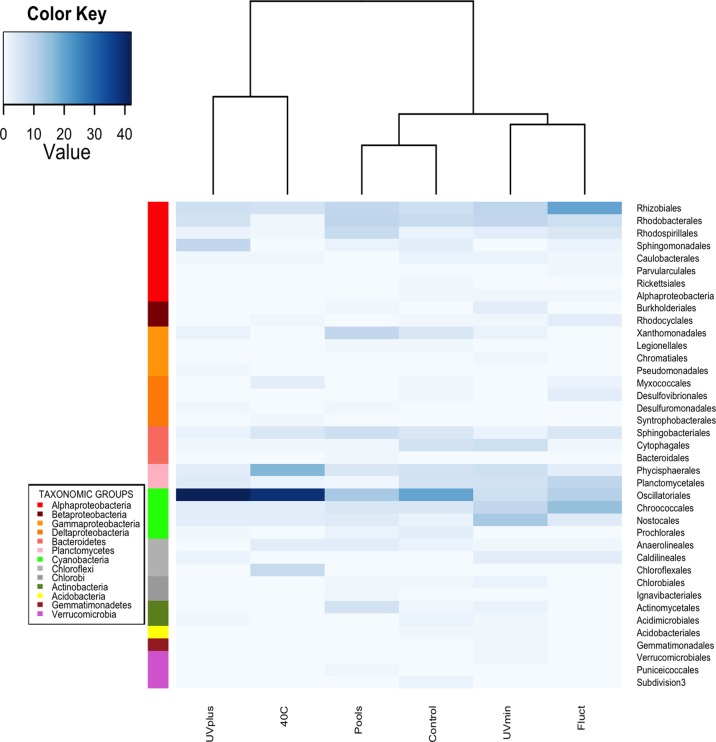
Heatmap showing the abundance of the 39 bacterial orders in the pools and treatments at the end of the experiment based on the 16S rRNA gene libraries. The Bray-Curtis dissimilarity matrix and Ward’s hierarchical clustering algorithm were applied in this analysis. Higher abundance is indicated by increased blue color intensity. The value shown in the color key scale represents the number of sequences detected for each environment. Rows indicate bacterial orders and columns indicate the different environments.

The clone libraries revealed that the bacterial mat communities from the less extreme treatments (Control and UVmin) had more similar compositions and distributions of the phylogenetic groups with the original pools than the most extreme treatments (UVplus, 40C and Fluct). These drastic changes in the community composition and structure indicated a different community response to each “press environmental disturbance” (Fig. 2). As expected, Control and UVmin were the treatments with the highest number of recovered bacterial OTUs (69 and 66 OTUs out of 98 and 104 sequences, respectively; Fig. 2 and Table 2). The most abundant orders in these treatments, as well as in the original pools, were Oscillatoriales, Rhodobacterales, and Rhizobiales, followed by Chroococcales, Cytophagales, Phycisphaerales, and Planctomycetales (Fig. 3). The Fluct treatment had a total of 52 OTUs out of 98 sequences, mainly from Rhizobiales, Chroococcales, Oscillatoriales, and Planctomycetales. Interestingly, the microbial mat seemed to resist the long-term fluctuating temperature better than the bacterioplankton community, which, over time, significantly decreased in OTUs number in this treatment [33]. We found the lowest number of OTUs in UVplus and 40C treatments (42 and 41 OTUs out of 93 and 97 sequences, respectively). The majority of sequences in the 40C treatment belonged to Oscillatoriales and Phycisphaerales, while the UVplus was largely dominated by one OTU from Oscillatoriales with close affinity with *Leptolyngbya*. This finding is similar to the bacterioplankton community [33], indicating that these latest treatments were the most different in community composition.

**Table 2:** Alpha diversity indices of the 16S rRNA gene sequences (OTUs at 97% cut-off) for each treatment and the natural pools.

	Sobs	Chao1	Shannon (H’)	Simpson (1/D)	Berger-Parker (BP)	Good’s coverage
Pools	60	129.2 (91.6–197.5)	3.91 (3.76–4.09)	0.015 (0.008–0.021)	0.075	58.2%
Control	69	228.6 (141.6–423.3)	4.02 (3.84–4.22)	0.017 (0.006–0.029)	0.11	47.1%
UVmin	66	182.9 (120.2–319.7)	3.93 (3.74–4.12)	0.019 (0.009–0.03)	0.085	51.9%
UVplus	42	76.4 (55.2–120.4)	3.01 (2.7–3.32)	0.115 (0.058–0.172)	0.338	69.1%
40C	41	176.3 (85.9–408.7)	3.21 (2.98–3.44)	0.065 (0.032–0.079)	0.165	70.2%
Fluct	52	101.9 (72.5–179.4)	3.63 (3.47–3.81)	0.029 (0.016–0.038)	0.122	64%

Confidence interval of each index in brackets; Sobs is the observed richness.

### Bacterial community diversity and structure

The Good’s coverage estimates, which were between 47.1 (Control) - 70.2% (40C), and the rarefaction curves based on the number of OTUs indicated that sampling was, as expected, not fully exhaustive, especially for the less stressful environments (Control and UVmin treatments and pools; Table 2 and S3 Fig.). Despite the fact that the bacterial community was insufficiently sampled, diversity indices showed that these less stressful environments had the highest bacterial diversity, while UVplus and 40C treatments strongly decreased bacterial evenness. In fact, a small number of OTUs with close affinity to *Leptolyngbya* dominated the microbial mat in both treatments (Table 1).

All treatments shared 10 bacterial orders out of 39 recovered orders with the original natural pools (Fig. 3); meanwhile, the majority of bacterial OTUs (80.1%) was unique to each treatment in the mesocosm experiment (S4 Fig.), which suggests that several rare taxa from the natural pools had an advantage in the different experimental conditions. However, despite the low sequencing depth, it is evident that the less extreme treatments (Control and UVmin) shared the largest number of OTUs (14 OTUs) and orders (19 orders), indicating that these environments maintain a more similar community than the more stressful environments.

**Fig 4:**
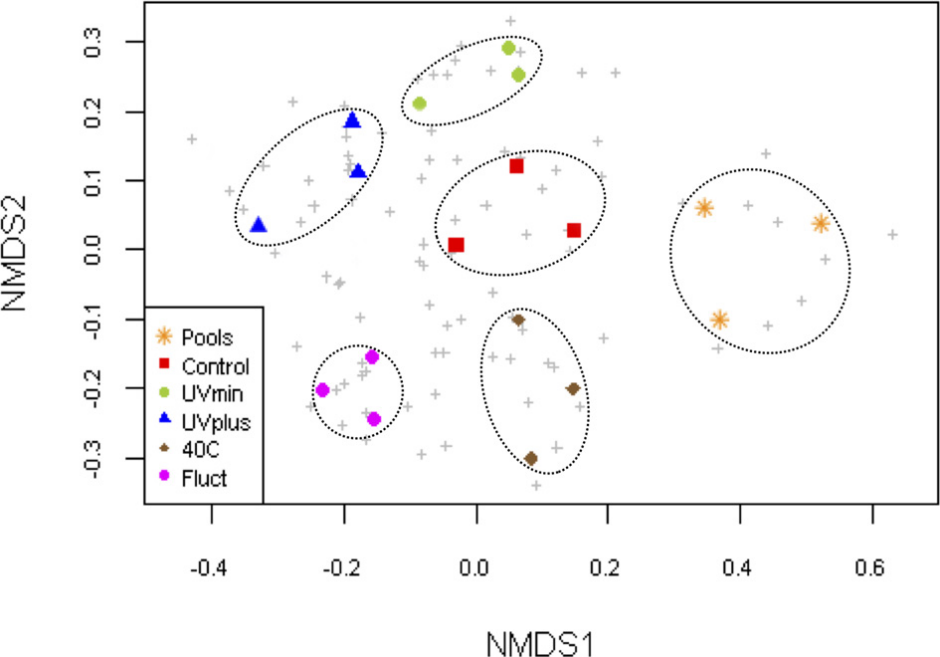
Non-metric multidimensional scaling (NMDS) ordination plot of Bray–Curtis community dissimilarities based on OTUs from the 16S rRNA gene sequences at the end of the experiment (2D stress value = 0.19). Color symbols represent the mesocosms from each treatment and pools, while grey crosses represent OTUs.

The NMDS plot separated the OTUs data into six distinct groups (Fig. 4), showing the strong grouping of the “microbial mat catchers” samples from the natural pools and the replicates of each treatment. This trend indicates replicability in the bacterial composition within each environment after long-term experimentation. We also confirmed this strong clustering trend with PERMANOVA and multivariate dispersion analyses (S2 Table), which revealed significant differences in bacterial composition among all environments (p< 0.001), even when considering only the treatments (p< 0.001), but not within them (pools and treatments: p = 0.79; only treatments: p = 0.61). All of these findings indicate that the treatments affected community composition and that long-term changes were replicated among mesocosms within each treatment. On the other hand, despite the fact that the bacterial mat composition was not analyzed at the beginning of this experiment, the cluster dendrogram showed that the bacterial community from the Control mesocosms was clustered within the untreated original pools and within the UVmin treatment groups after eight months of experimentation (S5 Fig.).

**Fig 5:**
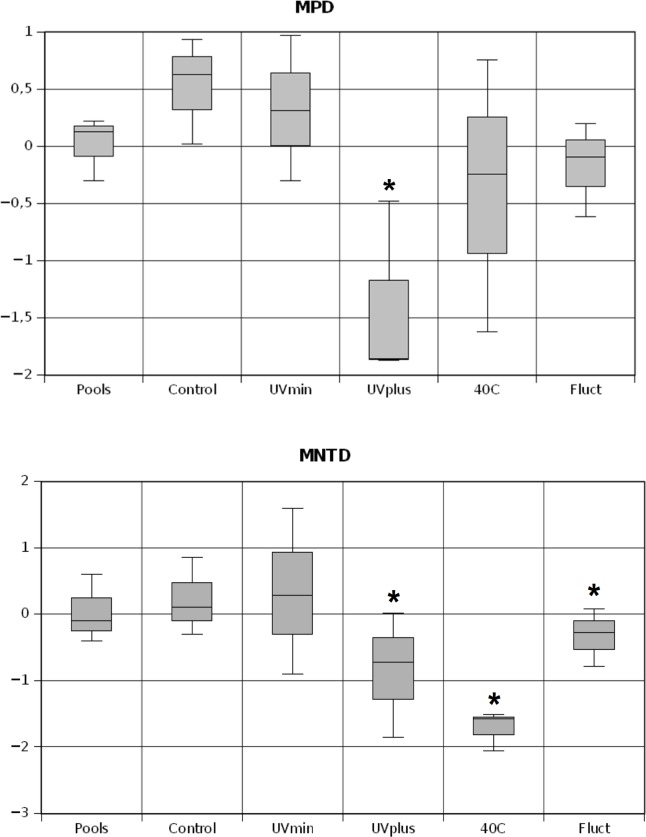
Box-and-whisker plots comparing the phylogenetic mean pairwise distance (MPD) and the mean nearest taxon distance (MNTD) values for the bacterial OTUs between pools and treatments. Asterisks indicate communities that are significantly structured at p< 0.05 level.

The phylogenetic community structure analyses pointed towards a random distribution of OTUs in the phylogeny for the Control and UVmin treatments as well as the pools at both MPD and MNTD metrics (Fig. 5). The UVplus treatment was significantly clustered in both metrics, indicating a closer phylogenetic relatedness (i.e., the coexistence of closely-related species) than expected by chance due to the strong environmental disturbance. Fluct and 40C treatments were significantly negative only for MNTD, which means that bacterial communities were composed of close relatives while displaying higher terminal phylogenetic clustering.

## Discussion

Global change, along with biodiversity erosion, threatens our planet as species move out of their ranges to follow niche requirements [52]. Microbes are the most ancient and important bioengineers of ecosystem functioning on the planet [5,6]. Thus, changes in microbial community composition due to environmental disturbances may directly affect ecosystem processes [53]. Our results have implications for understanding bacterial mat community responses to “press environmental disturbances”, such as those expected with global change (for example, increases in mean temperature or UV radiation).

To gain insight into bacterial community composition changes between different environments or conditions, it is useful to employ the strategies of whole-community DNA extraction, PCR amplification, cloning, and sequencing [54–56]. Similar to our previous work on bacterioplankton in the same mesocosms [33], we generated long rRNA sequences with Sanger sequence technology in order to identify organisms at a higher level of phylogenetic resolution. Shorter sequences produced by high-throughput next-generation sequencing methods usually do not contain sufficient taxonomic information, but have a much deeper coverage of the rare OTUs [57]. The sequences obtained from our gene libraries belong mainly to Cyanobacteria and Alphaproteobacteria phyla, which have been previously reported on microbial mats from the same pools [25] and stromatolites from CCB [47]. These phyla are also predominant and ubiquitous in microbial mats ecosystems [20,51,56,58,59]. This finding indicates that bacterial communities trapped in the “microbial mat catchers” from both the natural pools and the experimental treatments contained representatives of the original ecosystem. Furthermore, habitat affiliation confirmed the association of certain bacteria of this microbial mat community for similar environments. Hence, the 98% of sequences from the most abundant phylum (Cyanobacteria) was affiliated with freshwater, microbial mat, and hot spring habitats. The Rhizobiales, Sphingobacteriales, and Anerolineales orders were also associated with different freshwater and microbial mats ecosystems.

Due to confinement and handling effects, mesocosms experiments inherently introduce some bias in the evolution of bacterial communities compared to those naturally occurring in the field. However, these experimental tools are of great value in investigating how environmental disturbances and processes induce variations in bacterial populations [31,60]. In this long-term experiment, it is important to note that the community structure changes observed in triplicate mesocosms were reproducible within each treatment. Multivariate analyses confirmed the reproducibility of replicates and the differences between treatments, indicating that the distinct environments had a consistent effect on the development of particular bacterial assemblages. Not surprisingly, as a result of the mesocosms’ handling, even the bacterial mat community structure in the Control treatment deviated from the natural communities from the pools. Therefore, it would be better to compare data from mesocosms with the Control treatment and not directly with the original seed community. On the other hand, after eight months of experimentation, 10 bacterial orders from the natural pools were still present in all treatments (Fig. 3), suggesting that these organisms are generalists with ample tolerance to changes in UV and temperature. However, the majority of OTUs were unique to each treatment; for instance, members of Chloroflexales were only present in the 40C treatment. These filamentous anoxygenic phototrophs have been reported as dominant in hot spring microbial mats [61].

It has been suggested that environmental factors may influence the bacterial community composition at lower phylogenetic levels within major groups rather than changing the abundance at the phylum level [54]. This claim underscores the importance of assessing the underlying community composition rather than just the total diversity. This was the case for the phylum Planctomycetes, where two OTUs from Phycisphaeraceae and Planctomycetaceae significantly increased their abundance in the 40C and Fluct treatments, respectively. In addition, new OTUs were observed in the extreme treatments, such as Chloroflexales in 40C, OTUs belonging to Hyphomicrobiaceae in Fluct, and OTUs from Sphingomonadales in UVplus. If rare species were seeded from the natural pools, they should be rare under the same environmental conditions (Control treatment) and abundant when conditions become adequate for their growth [62].

Although cluster analysis showed that bacterial community structure from UVmin treatment resembled that from the Control treatment, we observed different OTUs in UVmin, most likely as a consequence of the increased community evenness in this treatment, allowing non-dominant groups to be sampled. On the other hand, the most drastic reduction in diversity was observed in the UVplus mesocosms, leading to the dominance of Cyanobacteria and Alphaproteobacteria clades that together contain 81.7% of the sampled community. Normally, when ambient UV intensity increases, photo-tolerant species will be selected. At the end of this experiment, certain Oscillatoriales OTUs, mainly from the genus *Leptolygbya*, increased their abundance in this treatment, indicating their high tolerance and acclimatization capacity for long-term UV exposure. It has been suggested that cyanobacteria microbial mats are resistant to the damaging effects of UV radiation by way of diverse mechanisms, such as escape through vertical migration, development of sunscreen pigments, quenching of reactive oxygen species, and damage-repair mechanisms [63].

Increased temperature (40C treatment) also affected community diversity through environmental filtering by increasing the dominance of a few adapted OTUs, such as heterotrophs from the Phycisphaeraceae family, and phototrophs from the Oscillatoriales (mainly the genus *Leptolygbya*) and Chloroflexales orders. These phototrophs are abundant in hot spring microbial mats [61], suggesting that they are adapted to high temperatures. On the other hand, high numbers of Planctomycetes have been reported in response to cyanobacterial blooms [64,65]. This is consistent with the chemoorganotrophic metabolism of Planctomycetes involved in degrading polymers produced by phototrophic organisms [66]. Thus, our results align with a bottom-up control of Planctomycetes via phototrophic production.

Temporal temperature fluctuations (Fluct treatment) affect the competitive outcome and would favor generalists that are well adapted to each alternating condition [67]. Diversity decreased for both the bacterioplankton [33] and bacterial mat communities in the fluctuating temperature treatment due to the low winter temperatures. However, the magnitude of the decrease differed, as the decrease in diversity was much greater in the bacterioplankton community [33]. Studies of microbial mats from different environments have shown that many of these microorganisms can adjust their pigment content and metabolism in order to optimize their growth over a broad temperature range [68,69]. Even though daily temperature remains almost constant in CCB pools, their microbial mats communities seem to be more able to adapt over time to this experiment’s sharply fluctuating temperature compared to the bacterioplankton community.

While the communities examined here were under-sampled, the use of a relative measure of community relatedness decreases the influence of under-sampling [8]. Phylogenetic metrics like MPD and MNTD are useful in investigating the ecological and evolutionary drivers of community assembly [11]. The phylogenetic clustering of OTUs in the most extreme treatments is consistent with the pattern expected if closely related taxa are ecologically similar [70], and these environments select for a limited subset of taxa that are able to survive and compete under stressful conditions. Therefore, phylogenetic metrics suggest that the long-term environmental disturbances in UVplus and 40C treatments constrained many OTUs, allowing only a few groups to survive and increase in abundance. This finding is consistent with a view suggesting that environmental disturbances in aquatic environments can result in assemblages that share many closely-related species [71]. To date, the relatively few studies that have quantitatively examined bacterial community structure in a phylogenetic framework suggest that such phylogenetic clustering may indicate the importance of environmental filtering in bacterial community assembly [8,9]. Furthermore, this phylogenetic clustering is particularly pronounced in stressful conditions, while overdispersion or neutral assembly usually occurs in more favorable conditions [8]. In line with this thinking, the phylogenetic indices show that the less stressful environments (Control and UVmin treatments) play a crucial role in the diversification and assembly of bacterial mat communities in this ancient oligotrophic ecosystem.

## Conclusions

If bacteria are the most important bioengineers on the planet, microbial mats and stromatolites are the “factory” assembling biogeochemical cycles since the Archaean. By studying the response of these communities to the environmental disturbances predicted under global change scenarios, we can test their stability against such alterations. Hence, we were able to explore the long-term effects of different environmental factors on bacterial mat communities’ composition and structure by combining a mesocosm experiment with multivariate and phylogenetic diversity analyses of molecular data.

This study shows that bacterial community composition within the microbial mats from CCB underwent a clear response to “press environmental disturbances” [29,30]. Bacterial diversity strongly decreased in the most extreme treatments, meaning that an increase in both temperature and UV irradiation causes a reduction of richness and increased dominance, with important implications for ecosystem functioning [72]. In addition, this study contributes relevant information on the role of certain environmental factors shaping bacterial mat community structure in this ancient oligotrophic ecosystem, which exhibits a different behavior from the associated planktonic bacterial communities. Moreover, it demonstrates how simple devices (glass slides submerged in pools) can retrieve part of the community composition of microbial mats. This mesocosm experiment thus allows us to manipulate and replicate aquatic ecosystems under different environmental scenarios, and in particular, to understand the sensibility of microbial mats communities to long-term environmental disturbances.

## Supporting Information

S1 Fig Neighbor-joining phylogenetic tree of 16S rRNA gene sequences from the microbial mats at the end of the experiment. The scale bar represents 1% estimated sequence divergence and the bold circles represents nodes that had >50% support in a bootstrap analysis of 1,000 replicates. The 250 OTUs from 600 sequences are defined at 0.03 distance cut-off. Clustered branches show the number of OTUs belonging to that group. OTU designations are followed (in parenthesis) by the number of sequences represented by that OTU in each environment. These designations are presented in the following order: Pools, Control, UVmin, UVplus, 40C, and Fluct.
(PDF)

S2 Fig Proportion of habitat affiliations of the representative bacterial OTUs of the microbial mats from the mesocosm experiment. It is based on the comparison of our 16S rRNA clone libraries sequences with their closest relatives in the Ribosomal Database Project using the Classifier tool.
(PDF)

S3 Fig Rarefaction curves showing 95% confidence interval of the 16S rRNA gene sequences for each environment at the end of the experiment. Curves display the number of OTUs detected (at 97% sequence identity) versus the number of sequences analysed in each environment.
(PDF)

S4 Fig Venn diagram illustrating shared (overlapping panels) and unique OTUs (non-overlapping panels) between the experimental treatments. The total OTUs number from each treatment is also indicated.
(PDF)

S5 Fig Differences of bacterial community structure between the natural pools and the mesocosms at the end of the experiment as determined by Canberra dissimilarity distances and the Ward’s hierarchical clustering algorithm. Cluster dendrogram is based on OTUs derived from the 16S rRNA gene libraries.
(PDF)

S1 Table Taxonomic assignment of the 250 representative OTUs (at 97% cut-off) of the microbial mats from the mesocosm experiment using the Ribosomal Database Project (RDP) tool for classification. OTU designations are followed (in parenthesis) by the number of sequences represented by that OTU in each environment with the following order: Pools, Control, UVmin, UVplus, 40C, and Fluct. The S_ab score represents the percentage of shared 7-mers between two sequences, which does not require the alignment for calculation. N° Seq is the number of sequences belonging to each OTU. Abbreviations: M is the mesocosm experiment; CCB is the Cuatro Cienegas Basin (Mexico); GN is Guerrero Negro (Mexico); A is Alchichica Lake (Mexico); Y is Yellowstone (USA).
(PDF)

S2 Table Results of Permutational multivariate analysis of variance (*adonis* function) and multivariate homogeneity of group dispersions analysis (*betadisper* function) of OTUs derived from 16S rRNA gene libraries data: a-b) between the different environments (pools and treatments); c-d) only between the treatments. Tests are based on Bray-Curtis dissimilarity distances and 999 permutations. P (MC): P value based on Monte Carlo random draws.
(PDF)
