# Supplementary figures and images for: Multivariate and Phylogenetic Analyses Assessing the Response of Bacterial Mat Communities from an Ancient Oligotrophic Aquatic Ecosystem to Different Scenarios of Long-Term Environmental Disturbance
Source: PLoS One. 2015 Mar 17;10(3):e0119741. doi: 10.1371/journal.pone.0119741 (PMC4363631; doi:10.1371/journal.pone.0119741)

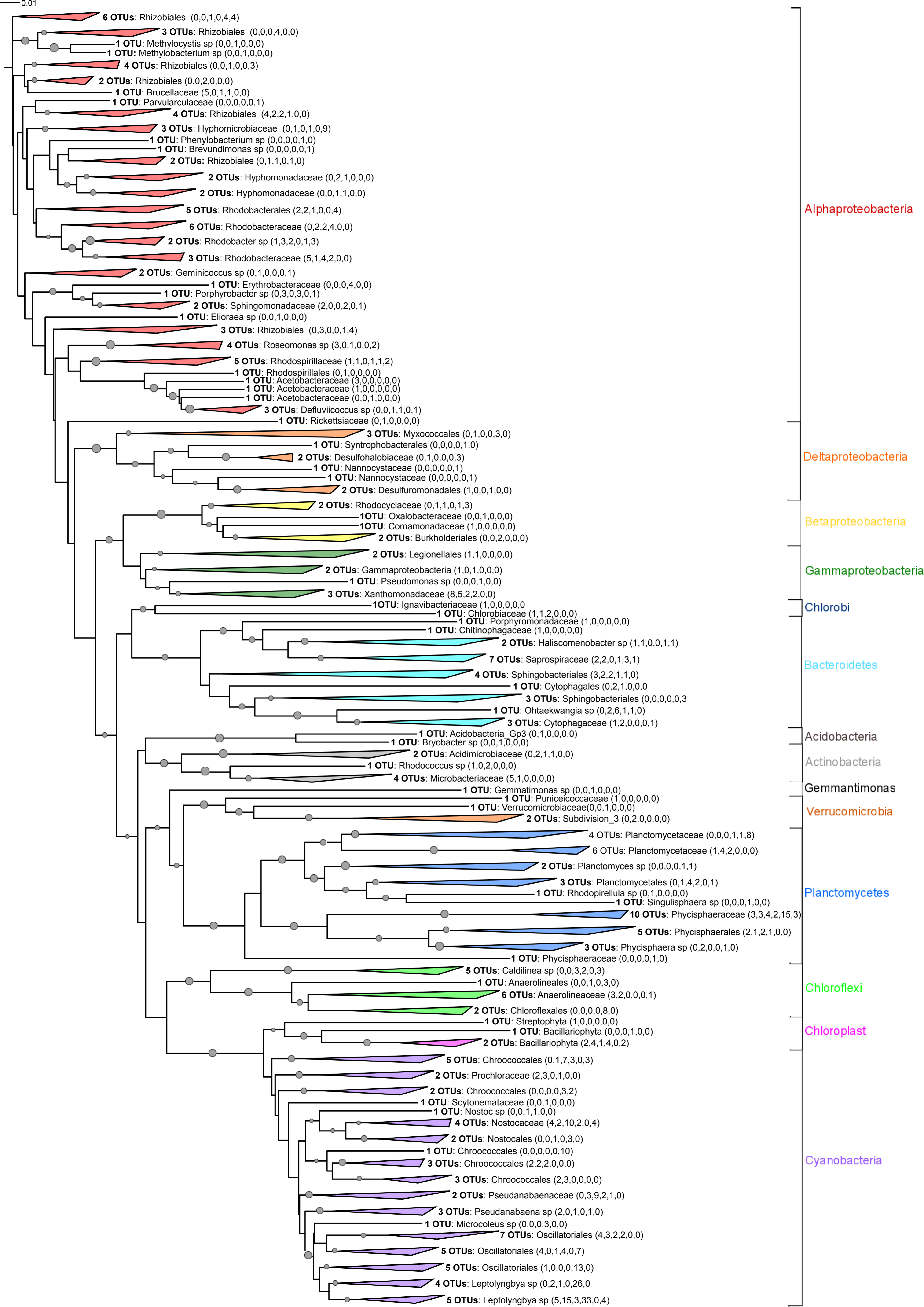

Supplement: S1 Fig — The scale bar represents 1% estimated sequence divergence and the bold circles represents nodes that had >50% support in a bootstrap analysis of 1,000 replicates. The 250 OTUs from 600 sequences are defined at 0.03 distance cut-off. Clustered branches show the number of OTUs belonging to that group. OTU designations are followed (in parenthesis) by the number of sequences represented by that OTU in each environment. These designations are presented in the following order: Pools, Control, UVmin, UVplus, 40C, and Fluct. (PDF) [file pone.0119741.s001.pdf]

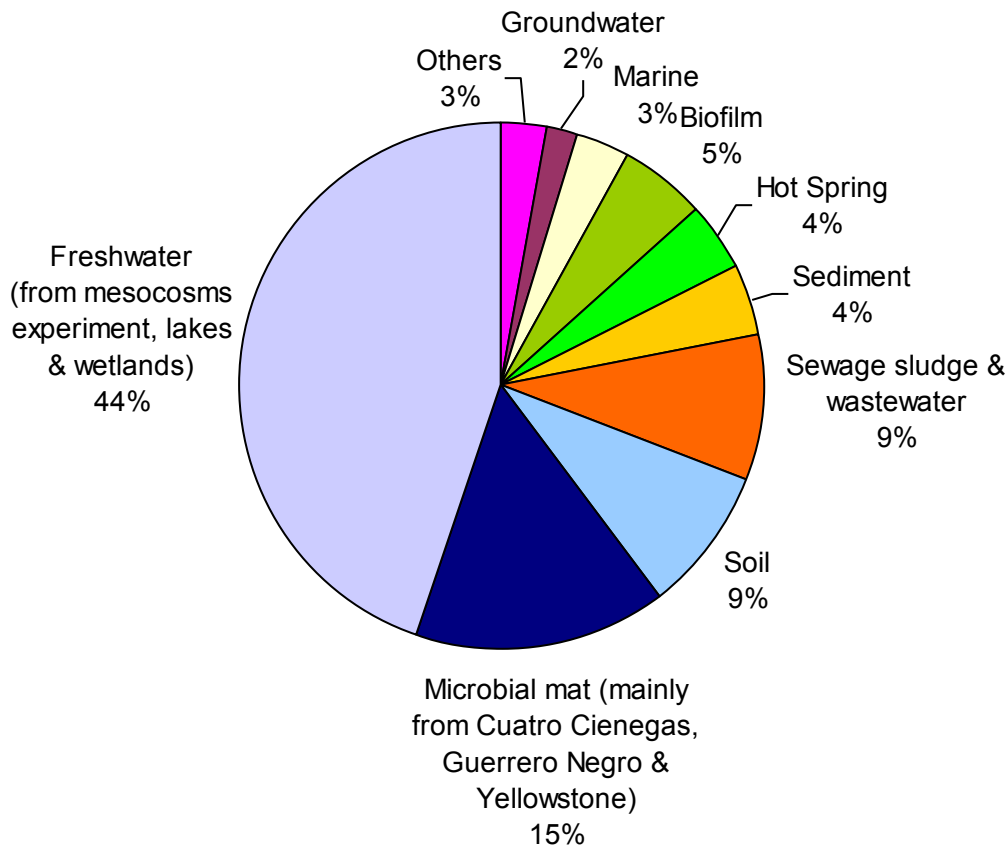

Supplement: S2 Fig — It is based on the comparison of our 16S rRNA clone libraries sequences with their closest relatives in the Ribosomal Database Project using the Classifier tool. (PDF) [file pone.0119741.s002.pdf]

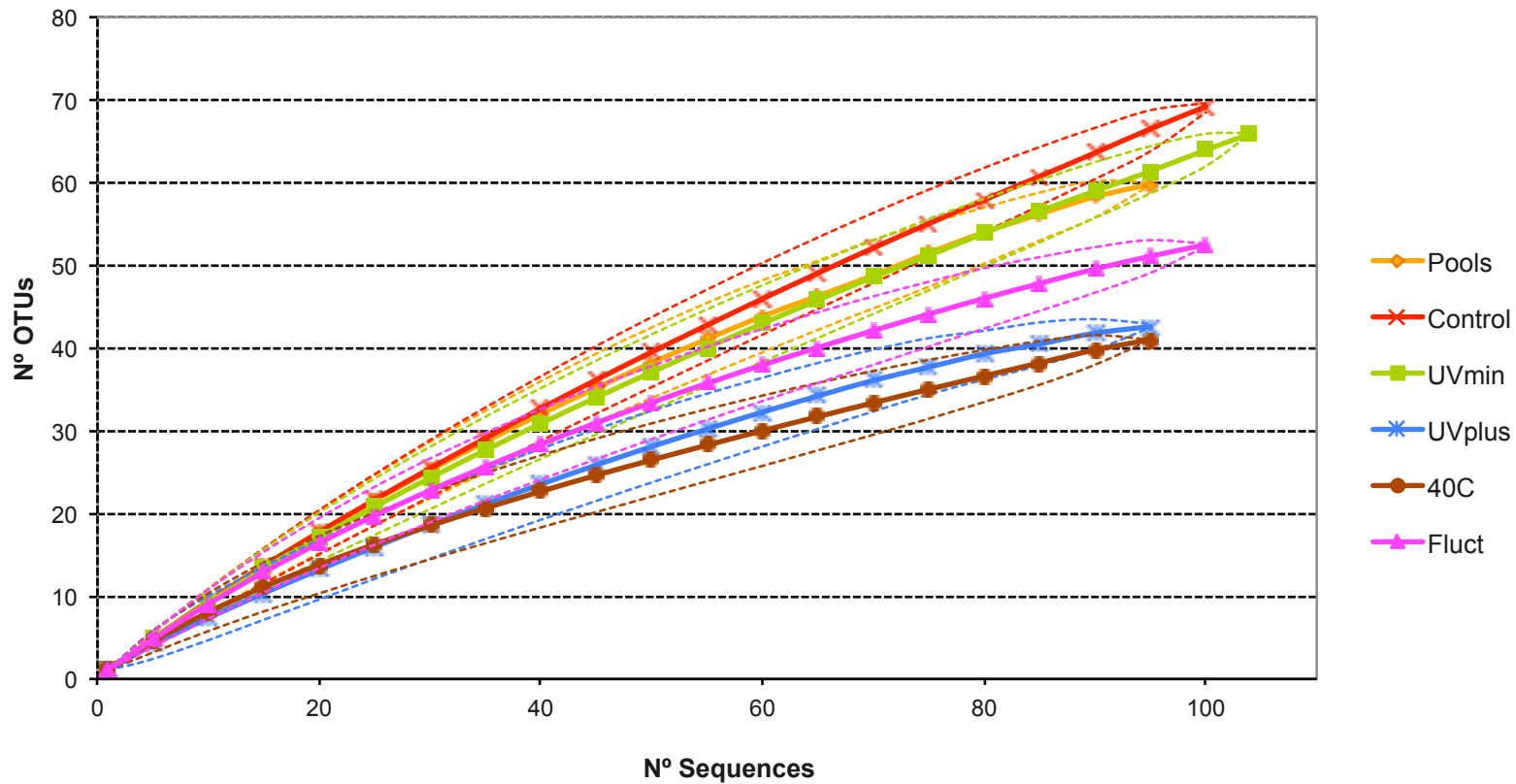

Supplement: S3 Fig — Curves display the number of OTUs detected (at 97% sequence identity) versus the number of sequences analysed in each environment. (PDF) [file pone.0119741.s003.pdf]

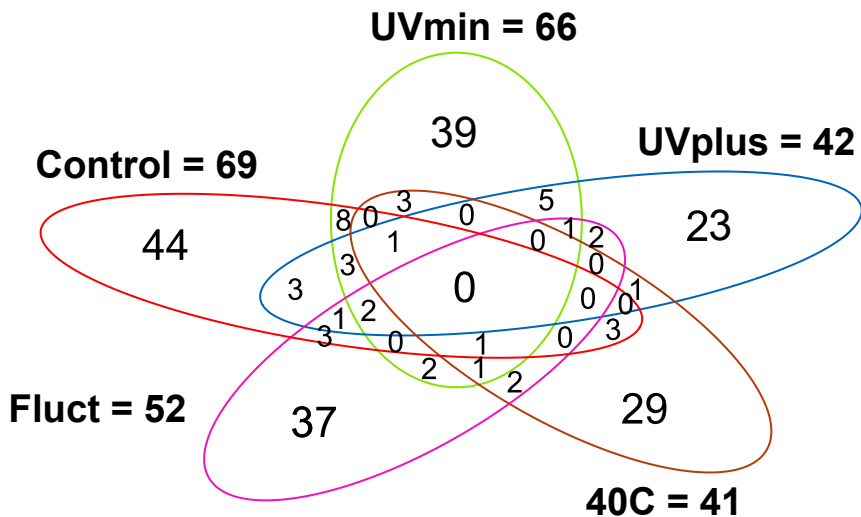

Supplement: S4 Fig — The total OTUs number from each treatment is also indicated. (PDF) [file pone.0119741.s004.pdf]

Canberra dissimilarity

0.7 0.9 1.1

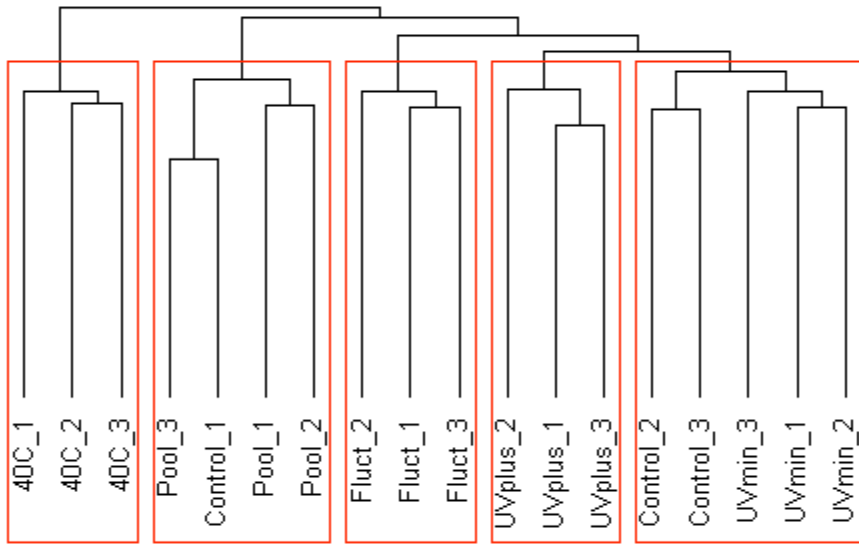

Pools and mesocomes  
hclust (\*, "ward")

Supplement: S5 Fig — Cluster dendrogram is based on OTUs derived from the 16S rRNA gene libraries. (PDF) [file pone.0119741.s005.pdf]
