# Supplementary material for: Multivariate and Phylogenetic Analyses Assessing the Response of Bacterial Mat Communities from an Ancient Oligotrophic Aquatic Ecosystem to Different Scenarios of Long-Term Environmental Disturbance
Source: PLoS One. 2015 Mar 17;10(3):e0119741. doi: 10.1371/journal.pone.0119741 (PMC4363631; doi:10.1371/journal.pone.0119741)
Supplement: S1 Table — OTU designations are followed (in parenthesis) by the number of sequences represented by that OTU in each environment with the following order: Pools, Control, UVmin, UVplus, 40C, and Fluct. The S_ab score represents the percentage of shared 7-mers between two sequences, which does not require the alignment for calculation. N° Seq is the number of sequences belonging to each OTU. Abbreviations: M is the mesocosm experiment; CCB is the Cuatro Cienegas Basin (Mexico); GN is Guerrero Negro (Mexico); A is Alchichica Lake (Mexico); Y is Yellowstone (USA). (PDF) [file pone.0119741.s006.pdf]

Table S1. Taxonomic assignment of the 250 representative OTUs (at 97% cut-off) of the microbial mats from the mesocosm experiment using the Ribosomal Database Project (RDP) tool for classification. OTU designations are followed (in parenthesis) by the number of sequences represented by that OTU in each environment with the following order: Pools, Control, UVmin, UVplus, 40C, and Fluct. The S_ab score represents the percentage of shared 7-mers between two sequences, which does not require the alignment for calculation. N° Seq is the number of sequences belonging to each OTU. Abbreviations: M is the mesocosm experiment; CCB is the Cuatro Cienegas Basin (Mexico); GN is Guerrero Negro (Mexico); A is Alchichica Lake (Mexico); Y is Yellowstone (USA).

OTU (97%)	Putative phylum	Best match sequence (RDP)	S_ab score	Habitat	N° Seq
Leptolyngbya sp (4,11,1,32,0,3)	Cyanobacteria	uncultured Leptolyngbya sp.; Cont2_11; JQ701207	1	Freshwater (M)	51
Leptolyngbya sp (0,2,0,0,16,0)	Cyanobacteria	uncultured Leptolyngbya sp.; 40C_3_32; JQ701237	0.982	Freshwater (M)	18
Nostocaceae bacterium (3,1,8,2,0,3)	Cyanobacteria	uncultured Nostocaceae cyanobacterium; UVmen2_20; JQ701223	0.973	Freshwater (M)	17
Pseudanabaenaceae bacterium (0,3,9,2,0,0)	Cyanobacteria	uncultured Leptolyngbya sp.; UVmen2_24; JQ701189	1	Freshwater (M)	14
Phycisphaeraceae bacterium (1,0,0,2,11,0)	Planctomycetes	uncultured Planctomycetales bacterium; 40C_3_23; JQ701181	0.92	Freshwater (M)	14
Stenotrophomonas sp (7,3,1,2,0,0)	Gammaproteobacteria	uncultured proteobacterium; YNP_BP_B75; DQ243741	1	Hot Springs (Y)	13
Rhodobacter sp (2,3,2,0,1,3)	Alphaproteobacteria	uncultured bacterium; P1CN01; AJ504474	0.941	Sewage sludge	11
Bacillariophyta (2,3,0,4,0,2)	Chloroplast	uncultured diatom; Cont2_4; JQ701252	1	Freshwater (M)	11
Ohtaekwangia sp (0,2,6,1,1,0)	Bacteroidetes	uncultured Cytophagaceae bacterium; 40C_2_31; JQ701691	0.99	Freshwater (M)	10
Chroococcales bacterium (0,0,0,0,0,10)	Cyanobacteria	uncultured cyanobacterium; SM2C12; AF445719	0.945	Hot Springs (Y)	10
Pseudanabaenaceae bacterium (1,0,0,0,9,0)	Cyanobacteria	uncultured Halomicronema sp.; Control_6; JQ701248	0.979	Freshwater (M)	10
Oscillatoriales bacterium (2,0,0,3,0,5)	Cyanobacteria	uncultured cyanobacterium; DGGE gel band MB-06-MIC-B2; EU882217	0.88	Lake	10
Brucellaceae bacterium (5,0,1,1,0,0)	Alphaproteobacteria	bacterium NWTT; HQ823639	1	Wetland	7

Hyphomicrobium sp (0,1,0,0,0,6)	Alphaproteobacteria	uncultured Hyphomicrobium sp.; Flu3_24; JQ701463	1	Freshwater (M)	7
Porphyrobacter sp (0,3,0,3,0,1)	Alphaproteobacteria	uncultured bacterium; 614; KC298380	0.906	Biofilm	7
Chroococcales bacterium (0,0,4,2,0,1)	Cyanobacteria	uncultured bacterium; BMB09; EF126218	0.944	Microbial mat	7
Planctomycetaceae bacterium (0,0,0,0,0,7)	Planctomycetes	uncultured bacterium; 3-4-51; JX012254	0.854	Groundwater	7
Rhodobacteraceae bacterium (0,0,4,2,0,0)	Alphaproteobacteria	uncultured Rhodobacter sp.; C1_28; JQ700882	1	Freshwater (M)	6
Leptolyngbya sp (0,0,0,0,6,0)	Cyanobacteria	Leptolyngbya sp. O-77; AB668059	0.98	Hot Springs	6
Planctomycetales bacterium (0,1,3,2,0,0)	Planctomycetes	unidentified bacterium; Sai4P3-84; AJ518752	0.876	Sediment	6
Rhodocyclaceae bacterium (0,0,1,0,1,3)	Betaproteobacteria	uncultured Zoogloea sp.; UVmen2_3; JQ701605	0.945	Freshwater (M)	5
Prochloraceae bacterium (2,3,0,0,0,0)	Cyanobacteria	uncultured bacterium; Layer1_G06; HQ882921	0.858	Microbialite (CCB)	5
Phycisphaeraceae bacterium (0,0,0,0,3,2)	Planctomycetes	uncultured bacterium; BACAP-OTU39; JQ791828	0.78	Biofilm	5
Erythrobacteraceae bacterium (0,0,0,4,0,0)	Alphaproteobacteria	uncultured bacterium; RT_S1_15; EU372619	0.868	Soil	4
Rhodobacteraceae bacterium (3,1,0,0,0,0)	Alphaproteobacteria	uncultured Labrenzia sp.; Fl1_12; JQ700901	0.831	Freshwater (M)	4
Sphingobacteriales bacterium (1,2,0,1,0,0)	Bacteroidetes	uncultured Runella sp.; Cont1_38; JQ701696	0.993	Freshwater (M)	4
Chlorobiaceae bacterium (1,1,2,0,0,0)	Chlorobi	uncultured Chlorobium sp.; UVmen3_23; JQ701146	1	Freshwater (M)	4
Anaerolineales bacterium (0,0,1,0,3,0)	Chloroflexi	uncultured Chloroflexi bacterium; UV-2_18; JQ700588	0.995	Freshwater (M)	4
Chloroflexales bacterium (0,0,0,0,4,0)	Chloroflexi	uncultured bacterium; Cyano2E11; EU917918	0.634	Microbialite	4
Chloroflexales bacterium (0,0,0,0,4,0)	Chloroflexi	uncultured Gram-positive bacterium; FL02D12; AY293435	0.709	Hot Springs (Y)	4
Leptolyngbya sp (0,0,0,0,4,0)	Cyanobacteria	Leptolyngbya sp. ISC 83; GU937790	0.773	Freshwater	4
Microbacteriaceae bacterium (3,0,0,0,0,0)	Actinobacteria	uncultured Microbacteriaceae bacterium; T0_32; JF413548	1	Freshwater (M)	3
Rhodococcus sp (1,0,2,0,0,0)	Actinobacteria	uncultured bacterium; N2_65J14f; AB484937	1	Soil	3
Acetobacteraceae bacterium (3,0,0,0,0,0)	Alphaproteobacteria	uncultured Acetobacteraceae bacterium; Flu2_24; JF413277	0.97	Freshwater (M)	3
Hyphomicrobium sp (0,0,0,0,0,3)	Alphaproteobacteria	uncultured bacterium; A58; JQ027092	0.96	Carbon filter	3
Rhizobiales bacterium (0,0,0,0,0,3)	Alphaproteobacteria	uncultured alpha proteobacterium; E06L-1; HE614839	0.782	Biofilm	3
Rhizobiales bacterium (0,1,0,0,0,2)	Alphaproteobacteria	uncultured bacterium; PA20; EU743866	0.729	Sewage sludge	3

Rhizobiales bacterium (0,2,0,0,1,0)	Alphaproteobacteria	uncultured Bradyrhizobiaceae bacterium; UVmen2_4; JQ701510	0.875	Freshwater (M)	3
Rhizobiales bacterium (1,2,0,0,0,0)	Alphaproteobacteria	uncultured bacterium; Layer2_B01; HQ882945	0.895	Microbialite (CCB)	3
Rhodobacterales bacterium (0,0,0,0,0,3)	Alphaproteobacteria	uncultured Bradyrhizobiaceae bacterium; UVmen2_4; JQ701510	0.941	Freshwater (M)	3
Roseomonas sp (2,0,0,0,0,1)	Alphaproteobacteria	uncultured bacterium; BSTSG-19; JN104841	0.876	River sediment	3
Sphingomonadaceae bacterium (2,0,0,0,0,1)	Alphaproteobacteria	uncultured bacterium; OX A10; FN429481	0.909	wastewater	3
Cytophagales bacterium (0,2,1,0,0,0)	Bacteroidetes	uncultured Chitinophaga sp.; Cont2_14; JQ701690	0.986	Freshwater (M)	3
Sphingobacteriales bacterium (0,0,2,0,1,0)	Bacteroidetes	uncultured bacterium; P060905_H09; HQ385626	0.957	Sewage sludge	3
Chroococcales bacterium (0,0,0,0,3,0)	Cyanobacteria	uncultured cyanobacterium; HAVOmat31; EF032786	0.751	Microbial mat	3
Chroococcales bacterium (0,0,0,1,0,2)	Cyanobacteria	uncultured cyanobacterium; DGGE gel band K7-7; DQ430970	0.953	Microbial mat	3
Chroococcales bacterium (2,1,0,0,0,0)	Cyanobacteria	Leptolyngbya sp. AECC1314; EU729072	0.945	Microbial mat	3
Leptolyngbya sp (0,3,0,0,0,0)	Cyanobacteria	uncultured Leptolyngbya sp.; Cont2_15; JQ701209	0.946	Freshwater (M)	3
Leptolyngbya sp (1,1,1,0,0,0)	Cyanobacteria	uncultured Leptolyngbya sp.; UVmen2_43; JQ701195	0.964	Freshwater (M)	3
Microcoleus sp (0,0,0,3,0,0)	Cyanobacteria	Geitlerinema sp. ZD; HQ197685	0.844	Hot Springs	3
Nostocaceae bacterium (0,0,0,0,3,0)	Cyanobacteria	uncultured Nostocaceae cyanobacterium; 40C_2_43; JQ701226	0.963	Freshwater (M)	3
Oscillatoriales bacterium (0,1,0,2,0,0)	Cyanobacteria	uncultured cyanobacterium; BCS92; EU728857	0.81	Microbial mat	3
Oscillatoriales bacterium (1,0,0,0,0,2)	Cyanobacteria	uncultured cyanobacterium; 17f04; GQ480644	0.956	Hot Springs	3
Oscillatoriales bacterium (3,0,0,0,0,0)	Cyanobacteria	uncultured cyanobacterium; DGGE band Ace-131b; EU009661	0.815	Microbial mat	3
Desulfovibrionales bacterium (0,1,0,0,0,2)	Deltaproteobacteria	uncultured bacterium; V201-33; HQ114048	0.922	Biofilm	3
Xanthomonadaceae bacterium (1,1,1,0,0,0)	Gammaproteobacteria	uncultured bacterium; P5; JQ958711	0.976	Lake	3
Acidimicrobiaceae bacterium (0,0,1,1,0,0)	Actinobacteria	uncultured actinobacterium; 27 WD64; EU447580	0.955	Sewage sludge	2
Ilumatobacter sp (0,2,0,0,0,0)	Actinobacteria	uncultured bacterium; LaP15L64; EF667674	0.929	River sediment	2
Hyphomonadaceae bacterium (0,2,0,0,0,0)	Alphaproteobacteria	uncultured alpha proteobacterium; Alchichica_AQ1_2_1B_181; JN825359	0.929	Lake (A)	2

Rhizobiales bacterium (0,0,0,2,0,0)	Alphaproteobacteria	uncultured Rhizobiales bacterium; UVmas3_51; JF413750	0.969	Freshwater (M)	2
Rhizobiales bacterium (0,0,1,0,0,1)	Alphaproteobacteria	uncultured Phyllobacteriaceae bacterium; UVmen1_4; JQ701534	0.803	Freshwater (M)	2
Rhizobiales bacterium (0,0,1,0,1,0)	Alphaproteobacteria	uncultured Rhizobiaceae bacterium; 40_1_36; JF412861	0.979	Freshwater (M)	2
Rhizobiales bacterium (0,0,2,0,0,0)	Alphaproteobacteria	uncultured bacterium; C-131; KC836050	0.996	Marine	2
Rhizobiales bacterium (1,0,0,1,0,0)	Alphaproteobacteria	uncultured bacterium; kab196; FJ936913	0.957	Mud volvano	2
Rhizobiales bacterium (2,0,0,0,0,0)	Alphaproteobacteria	uncultured Phyllobacteriaceae bacterium; UVmen1_4; JQ701534	0.954	Freshwater (M)	2
Rhodobacteraceae bacterium (0,0,0,2,0,0)	Alphaproteobacteria	uncultured bacterium; TFAgeg181; HE659150	0.869	Sediment	2
Rhodobacteraceae bacterium (0,1,1,0,0,0)	Alphaproteobacteria	uncultured Rhodobacteraceae bacterium; UVmen3_16; JQ701671	0.964	Freshwater (M)	2
Rhodobacteraceae bacterium (2,0,0,0,0,0)	Alphaproteobacteria	uncultured alpha proteobacterium; M-22; AM229524	0.945	Microbial mat (Y)	2
Rhodobacterales bacterium (0,1,1,0,0,0)	Alphaproteobacteria	uncultured bacterium; 614; KC298380	0.906	Biofilm	2
Rhodobacterales bacterium (2,0,0,0,0,0)	Alphaproteobacteria	uncultured alpha proteobacterium; A23YA11RM; FJ569291	0.764	Soil	2
Rhodobiaceae bacterium (0,0,0,0,0,2)	Alphaproteobacteria	uncultured bacterium; CG10; AB491708	0.703	Hot Springs	2
Rhodospirillaceae bacterium (1,1,0,0,0,0)	Alphaproteobacteria	uncultured Phyllobacteriaceae bacterium; Cont3_31; JQ701642	0.99	Freshwater (M)	2
Sphingomonadaceae bacterium (0,0,0,2,0,0)	Alphaproteobacteria	uncultured Sphingopyxis sp.; UVmas2_43; JQ701429	0.929	Freshwater (M)	2
Cytophagaceae bacterium (1,1,0,0,0,0)	Bacteroidetes	uncultured bacterium; Layer1_H06; HQ882932	0.769	Microbialite (CCB)	2
Haliscomenobacter sp (0,0,0,0,1,1)	Bacteroidetes	uncultured bacterium; Layer1_C05; HQ882876	0.881	Microbialite (CCB)	2
Haliscomenobacter sp (1,1,0,0,0,0)	Bacteroidetes	uncultured bacterium; PA69; EU743894	0.89	Sewage sludge	2
Saprospiraceae bacterium (0,1,0,0,1,0)	Bacteroidetes	uncultured Saprospiraceae bacterium; Cont1_61; JF413097	0.991	Freshwater (M)	2
Saprospiraceae bacterium (2,0,0,0,0,0)	Bacteroidetes	uncultured bacterium; V201-175; HQ114190	0.794	Biofilm	2
Caldilinea sp (0,0,0,0,0,2)	Chloroflexi	uncultured bacterium; BJGMM-1s-310; JQ800792	0.745	Soil	2
Caldilinea sp (0,0,1,1,0,0)	Chloroflexi	uncultured bacterium; 31; FJ623297	0.852	Sewage sludge	2
Caldilinea sp (0,0,2,0,0,0)	Chloroflexi	uncultured Chloroflexi bacterium; UVmen3_35;	1	Freshwater (M)	2

		JQ701156			
Bacillariophyta (0,1,1,0,0,0)	Chloroplast	uncultured cyanobacterium; CYAVE50; FJ774032	0.852	Lake	2
Chroococcales bacterium t (0,0,0,0,0,2)	Cyanobacteria	uncultured Prochlorothrix sp.; Cont2_29; JQ701194	0.903	Freshwater (M)	2
Chroococcales bacterium (0,0,2,0,0,0)	Cyanobacteria	uncultured bacterium; B0610R001_H23; AB657800	0.759	Soil	2
Chroococcales bacterium (0,0,2,0,0,0)	Cyanobacteria	uncultured Leptolyngbya sp.; UVmen2_25; JQ701197	0.764	Freshwater (M)	2
Chroococcales bacterium (1,1,0,0,0,0)	Cyanobacteria	uncultured Chroococcales cyanobacterium; Cont3_4; JF413184	0.991	Freshwater (M)	2
Synechococcaceae bacterium (2,0,0,0,0,0)	Cyanobacteria	uncultured bacterium; B-35; HQ661201	1	Freshwater	2
Leptolyngbya sp (0,0,1,0,0,1)	Cyanobacteria	uncultured bacterium; C1_12; JQ700608	0.99	Freshwater (M)	2
Nostoc sp (0,0,1,1,0,0)	Cyanobacteria	uncultured cyanobacterium; UVmen1_37; JQ701169	1	Freshwater (M)	2
Nostocaceae bacterium (0,0,2,0,0,0)	Cyanobacteria	uncultured cyanobacterium; DGGE gel band MB-06-MIC-B13; EU882221	0.891	Lake	2
Nostocaceae bacterium (1,0,0,0,0,1)	Cyanobacteria	uncultured Nostocaceae cyanobacterium; Flu1_18; JQ701162	1	Freshwater (M)	2
Pseudanabaena sp (2,0,0,0,0,0)	Cyanobacteria	uncultured Oscillatoriales cyanobacterium; Flu2_7; JF413310	0.936	Freshwater (M)	2
Myxococcales bacterium (0,0,0,0,2,0)	Deltaproteobacteria	uncultured delta proteobacterium; Alchichica_AQ2_1_1B_12; JN825520	0.76	Lake (A)	2
Phycisphaeraceae bacterium (0,0,2,0,0,0)	Planctomycetes	uncultured Planctomycetales bacterium; UVmen3_38; JQ701685	0.97	Freshwater (M)	2
Phycisphaeraceae bacterium (0,1,1,0,0,0)	Planctomycetes	uncultured bacterium; Be_021; FR667403	0.647	Biofilm	2
Phycisphaeraceae bacterium (1,0,0,0,0,1)	Planctomycetes	uncultured deep-sea bacterium; Ucs1505; AM997706	0.697	Sea sediment	2
Phycisphaerales bacterium (0,1,0,1,0,0)	Planctomycetes	uncultured bacterium; BMT05; EF126245	0.913	Microbial mat	2
Planctomycetaceae bacterium (1,1,0,0,0,0)	Planctomycetes	uncultured Pirellula sp.; CL2.C40; FM175380	0.979	Freshwater	2
Acidobacteria_Gp3 bacterium (0,1,0,0,0,0)	Acidobacteria	uncultured bacterium; F5-B48; FJ206864	0.823	Microbial mat (Y)	1
Bryobacter sp (0,0,1,0,0,0)	Acidobacteria	uncultured bacterium; Reactor5_71; JQ629895	0.865	Bioreactor	1
Microbacteriaceae bacterium (0,1,0,0,0,0)	Actinobacteria	uncultured actinobacterium; c-10; GQ144943	0.955	Marine	1
Microbacteriaceae bacterium (1,0,0,0,0,0)	Actinobacteria	uncultured actinobacterium; Ume_OR_81; FJ646424	0.903	Freshwater	1

Microbacteriaceae bacterium (1,0,0,0,0,0)	Actinobacteria	uncultured Actinobacteridae bacterium; UVmas3_86; JF413782	1	Freshwater (M)	1
Acetobacteraceae bacterium (0,0,1,0,0,0)	Alphaproteobacteria	Uncultured bacterium A11; CW3P1_7F; KC110528	0.833	Wastewater	1
Acetobacteraceae bacterium (1,0,0,0,0,0)	Alphaproteobacteria	uncultured bacterium; F5K2Q4C04IXZNE; GU915450	0.84	Sewage sludge	1
Brevundimonas sp (0,0,0,0,0,1)	Alphaproteobacteria	uncultured bacterium; XYHPA.0912.106; HQ904741	0.982	Lake	1
Defluviicoccus sp (0,0,0,0,0,1)	Alphaproteobacteria	uncultured bacterium; Layer2_D06; HQ882971	0.93	Microbialite (CCB)	1
Defluviicoccus sp (0,0,0,1,0,0)	Alphaproteobacteria	uncultured organism; W-OTU3; JN638452	0.893	Sewage sludge	1
Defluviicoccus sp (0,0,1,0,0,0)	Alphaproteobacteria	uncultured cyanobacterium; DGGE gel band MB-06-MIC-B11; EU882218	0.777	Lake	1
Elioraea sp (0,0,1,0,0,0)	Alphaproteobacteria	uncultured bacterium; F5-B11; FJ206847	0.969	Microbial mat (Y)	1
Geminicoccus sp (0,0,0,0,0,1)	Alphaproteobacteria	uncultured alpha proteobacterium; Alchichica_AQ1_1_1B_74; JN825372	0.84	Lake (A)	1
Geminicoccus sp (0,1,0,0,0,0)	Alphaproteobacteria	uncultured bacterium; 293c2; EF460011	0.727	Sea sediment	1
Hyphomicrobiaceae bacterium (0,0,0,1,0,0)	Alphaproteobacteria	uncultured bacterium; F7-B72; FJ206967	0.893	Microbial mat (Y)	1
Hyphomonadaceae bacterium (0,0,0,1,0,0)	Alphaproteobacteria	uncultured organism; SBZP_3756; JN537099	0.797	Microbial mat (GN)	1
Hyphomonadaceae bacterium (0,0,1,0,0,0)	Alphaproteobacteria	uncultured Ochrobactrum sp.; C2_33; JQ700800	1	Freshwater (M)	1
Hyphomonas sp (0,0,1,0,0,0)	Alphaproteobacteria	uncultured Hyphomonas sp.; C1_31; JQ700784	0.99	Freshwater (M)	1
Methylobacterium sp (0,0,1,0,0,0)	Alphaproteobacteria	Methylobacterium sp. DDW-1; FJ225120	0.979	Soil	1
Methylocystis sp (0,0,1,0,0,0)	Alphaproteobacteria	uncultured proteobacterium; DGGE band 9alpha 6; AJ429168	0.913	Soil	1
Parvularculaceae bacterium (0,0,0,0,0,1)	Alphaproteobacteria	uncultured alpha proteobacterium; F10CONTROL D35; GQ242877	0.784	Sea sediment	1
Phenylobacterium sp (0,0,0,0,1,0)	Alphaproteobacteria	uncultured Phenylobacterium sp.; 40C_1_35; JQ701304	1	Freshwater (M)	1
Rhizobiaceae bacterium (0,0,0,1,0,0)	Alphaproteobacteria	uncultured Rhizobiaceae bacterium; T0_17; JF413471	0.97	Freshwater (M)	1
Rhizobiales bacterium (0,0,0,0,0,1)	Alphaproteobacteria	uncultured bacterium; sw019; GQ258096	0.76	Marine	1
Rhizobiales bacterium (0,0,0,0,0,1)	Alphaproteobacteria	uncultured organism; SBZA_e704; JN499892	0.765	Microbial mat (GN)	1

Rhizobiales bacterium (0,0,0,0,0,1)	Alphaproteobacteria	uncultured Phyllobacteriaceae bacterium; UVmen1_15; JQ701540	0.989	Freshwater (M)	1
Rhizobiales bacterium (0,0,0,0,1,0)	Alphaproteobacteria	uncultured bacterium; DP7.3.50; FJ612219	0.816	Lake	1
Rhizobiales bacterium (0,0,0,0,1,0)	Alphaproteobacteria	uncultured Labrenzia sp.; 40-3_23; JQ700900	0.822	Freshwater (M)	1
Rhizobiales bacterium (0,0,0,0,1,0)	Alphaproteobacteria	uncultured bacterium; BRA041; EU284313	0.875	Sediment	1
Rhizobiales bacterium (0,0,0,0,1,0)	Alphaproteobacteria	uncultured Parvibaculum sp.; 40C_3_13; JQ701587	0.952	Freshwater (M)	1
Rhizobiales bacterium (0,0,0,1,0,0)	Alphaproteobacteria	uncultured Mesorhizobium sp.; UVmas1_55; JQ701343	0.989	Freshwater (M)	1
Rhizobiales bacterium (0,0,1,0,0,0)	Alphaproteobacteria	uncultured bacterium; SW-6A_G08; JX286074	0.722	Drinking water	1
Rhizobiales bacterium (0,0,1,0,0,0)	Alphaproteobacteria	uncultured bacterium; HB9; AY996569	0.845	Groundwater	1
Rhizobiales bacterium (0,0,1,0,0,0)	Alphaproteobacteria	uncultured bacterium; 300C8; JF827556	0.876	Marine	1
Rhizobiales bacterium (0,1,0,0,0,0)	Alphaproteobacteria	uncultured organism; SBZC_1386; JN505561	0.855	Microbial mat (GN)	1
Rhodobacter sp (1,0,0,0,0,0)	Alphaproteobacteria	uncultured bacterium; P1CN01; AJ504474	0.831	Sewage sludge	1
Rhodobacteraceae bacterium (0,0,0,1,0,0)	Alphaproteobacteria	uncultured bacterium; PA20; EU743866	0.826	Sewage sludge	1
Rhodobacteraceae bacterium (0,0,0,1,0,0)	Alphaproteobacteria	uncultured bacterium; Baqar.Sed.Eubac.20; AB355069	0.959	Sediment	1
Rhodobacteraceae bacterium (0,0,1,0,0,0)	Alphaproteobacteria	uncultured alpha proteobacterium; PA-C40; DQ295412	0.704	Soil	1
Rhodobacteraceae bacterium (0,1,0,0,0,0)	Alphaproteobacteria	uncultured bacterium; PA20; EU743866	0.766	Sewage sludge	1
Rhodobacterales bacterium (0,0,0,0,0,1)	Alphaproteobacteria	uncultured alpha proteobacterium; WT172G01R; EU079363	0.807	Rhizoplane	1
Rhodobacterales bacterium (0,1,0,0,0,0)	Alphaproteobacteria	uncultured Rhodobacterales bacterium; C3_20; JQ700856	0.949	Freshwater (M)	1
Rhodospirillaceae bacterium (0,0,0,0,0,1)	Alphaproteobacteria	uncultured bacterium; S12_025; KC872142	0.816	Marine	1
Rhodospirillaceae bacterium (0,0,0,0,0,1)	Alphaproteobacteria	uncultured bacterium; DH100507_12B; KC358506	0.852	Groundwater	1
Rhodospirillaceae bacterium (0,0,0,0,1,0)	Alphaproteobacteria	uncultured Rhodocista sp.; 40_2_26; JF412910	0.968	Freshwater (M)	1
Rhodospirillaceae bacterium (0,0,0,1,0,0)	Alphaproteobacteria	uncultured bacterium; Layer2_G05; HQ883003	0.886	Microbialite (CCB)	1
Rhodospirillales bacterium (0,1,0,0,0,0)	Alphaproteobacteria	uncultured bacterium; sw019; GQ258096	0.664	Marine	1
Rickettsiaceae bacterium (0,1,0,0,0,0)	Alphaproteobacteria	uncultured cyanobacterium; DGGE gel band MB-06-MIC-B11; EU882218	0.705	Lake	1

Roseomonas sp (0,0,0,0,0,1)	Alphaproteobacteria	uncultured alpha proteobacterium; AKYG679; AY922054	0.823	Soil	1
Roseomonas sp (0,0,1,0,0,0)	Alphaproteobacteria	uncultured Roseomonas sp.; 40C_2_19; JQ701648	0.887	Freshwater (M)	1
Roseomonas sp (1,0,0,0,0,0)	Alphaproteobacteria	uncultured alpha proteobacterium; M1_F10; EU051946	0.818	Soil	1
Chitinophagaceae bacterium (1,0,0,0,0,0)	Bacteroidetes	uncultured Sphingobacteriales bacterium; T0_110; JF413409	0.981	Freshwater (M)	1
Cytophagaceae bacterium (0,0,0,0,0,1)	Bacteroidetes	Uncultured bacterium; Layer1_E08; HQ882899	0.631	Microbialite (CCB)	1
Cytophagaceae bacterium (0,1,0,0,0,0)	Bacteroidetes	Algoriphagus aquatilis; NBRC 104237; AB682160	0.848	Lake	1
Porphyromonadaceae bacterium (1,0,0,0,0,0)	Bacteroidetes	uncultured Rikenellaceae bacterium; T0_46; JF413562	1	Freshwater (M)	1
Saprospiraceae bacterium (0,0,0,0,0,1)	Bacteroidetes	uncultured bacterium; SINI968; HM127278	0.783	Lake	1
Saprospiraceae bacterium (0,0,0,0,1,0)	Bacteroidetes	uncultured Bacteroidetes bacterium; DGGE band GWS-AG-9; AY274243	0.625	Marine	1
Saprospiraceae bacterium (0,0,0,0,1,0)	Bacteroidetes	uncultured organism; MAT-CR-H3-E04; EU245170	0.729	Microbial mat (Y)	1
Saprospiraceae bacterium (0,0,0,1,0,0)	Bacteroidetes	uncultured organism; SBYX_5997; JN490935	0.695	Microbial mat (GN)	1
Saprospiraceae bacterium (0,1,0,0,0,0)	Bacteroidetes	uncultured Lewinella sp.; Cont2_28; JQ701693	1	Freshwater (M)	1
Sphingobacteriales bacterium (0,0,0,0,0,1)	Bacteroidetes	uncultured bacterium; DGGE Band 11; AY487128	0.684	Biofilm	1
Sphingobacteriales bacterium (0,0,0,0,0,1)	Bacteroidetes	uncultured bacterium; Layer2_G06; HQ883004	0.908	Microbialite (CCB)	1
Sphingobacteriales bacterium (0,0,0,0,0,1)	Bacteroidetes	uncultured bacterium; Layer2_E03; HQ882980	0.937	Microbialite (CCB)	1
Sphingobacteriales bacterium (1,0,0,0,0,0)	Bacteroidetes	uncultured bacterium; Layer1_D12; HQ882891	0.669	Microbialite (CCB)	1
Sphingobacteriales bacterium (1,0,0,0,0,0)	Bacteroidetes	uncultured bacterium; Layer1_B09; HQ882869	0.8	Microbialite (CCB)	1
Burkholderiales bacterium (0,0,1,0,0,0)	Betaproteobacteria	uncultured bacterium; F5K2Q4C04IOOCS; GU916301	0.847	Sewage sludge	1
Comamonadaceae bacterium (0,0,1,0,0,0)	Betaproteobacteria	uncultured Rhodoferax sp.; SSCP Band 10; AM267489	0.785	Biofilm	1
Comamonadaceae bacterium (1,0,0,0,0,0)	Betaproteobacteria	uncultured bacterium; PA31; EU743899	0.959	Sewage sludge	1
Methyloversatilis sp (0,1,0,0,0,0)	Betaproteobacteria	uncultured bacterium; ZBAF3-24; HQ682050	0.952	Biofilm	1

Oxalobacteraceae bacterium (0,0,1,0,0,0)	Betaproteobacteria	uncultured Ralstonia sp.; UVmen3_40; JQ701607	0.98	Freshwater (M)	1
Ignavibacteriaceae bacterium (1,0,0,0,0,0)	Chlorobi	uncultured bacterium; SG102-3_25; HM146701	0.761	Sediment	1
Anaerolineaceae bacterium (0,0,0,0,0,1)	Chloroflexi	uncultured organism; SBYN_2222; JN461990	0.731	Microbial mat (GN)	1
Anaerolineaceae bacterium (0,1,0,0,0,0)	Chloroflexi	uncultured organism; SBXY_5453; JN430136	0.71	Microbial mat (GN)	1
Anaerolineaceae bacterium (0,1,0,0,0,0)	Chloroflexi	uncultured organism; SBYT_5140; JN482597	0.719	Microbial mat (GN)	1
Anaerolineaceae bacterium (1,0,0,0,0,0)	Chloroflexi	uncultured organism; SBXZ_3827; JN433121	0.579	Microbial mat (GN)	1
Anaerolineaceae bacterium (1,0,0,0,0,0)	Chloroflexi	uncultured Chloroflexi bacterium; TDNP_Bbc97_242_1_63; FJ516783	0.677	Freshwater	1
Anaerolineaceae bacterium (1,0,0,0,0,0)	Chloroflexi	uncultured Chloroflexi bacterium; IAFpp7112; GU214126	0.708	wastewater	1
Caldilinea sp (0,0,0,0,0,1)	Chloroflexi	uncultured bacterium; F5K2Q4C04IB9N9; GU913326	0.803	Sewage sludge	1
Caldilinea sp (0,0,0,1,0,0)	Chloroflexi	uncultured bacterium; NBBAB0409_66; JQ072889	0.936	Sewage sludge	1
Bacillariophyta (0,0,0,1,0,0)	Chloroplast	uncultured bacterium; Layer2_D06; HQ882971	0.93	Microbialite (CCB)	1
Streptophyta (1,0,0,0,0,0)	Chloroplast	Glechoma hederacea; DQ417652	0.987	Soil	1
Chroococcales bacterium (0,0,1,0,0,0)	Cyanobacteria	uncultured cyanobacterium; DGGE gel band MB-06-MIC-B2; EU882217	0.908	Lake	1
Chroococcales bacterium (0,1,0,0,0,0)	Cyanobacteria	uncultured cyanobacterium; DGGE gel band K7-7; DQ430970	0.766	Microbial mat	1
Chroococcales bacterium (0,1,0,0,0,0)	Cyanobacteria	uncultured cyanobacterium; DGGE gel band MB-06-MIC-B2; EU882217	0.826	Lake	1
Chroococcales bacterium (0,1,0,0,0,0)	Cyanobacteria	uncultured cyanobacterium; BAC_2_H01; FJ967935	0.858	Hot Springs	1
Leptolyngbya sp (0,0,0,1,0,0)	Cyanobacteria	uncultured Leptolyngbya sp.; UVmas1_54; JQ701193	1	Freshwater (M)	1
Leptolyngbya sp (0,0,1,0,0,0)	Cyanobacteria	uncultured Leptolyngbya sp.; UVmen2_25; JQ701197	0.961	Freshwater (M)	1
Nostocaceae bacterium (0,1,0,0,0,0)	Cyanobacteria	cyanobacterium LEGE 06123; FJ589716	0.928	Marine	1
Nostocales bacterium (0,0,1,0,0,0)	Cyanobacteria	uncultured cyanobacterium; DGGE gel band MB-06-MIC-B2; EU882217	0.87	Lake	1
Oscillatoriales bacterium (0,0,0,0,1,0)	Cyanobacteria	uncultured cyanobacterium; TDNP_Wbc97_251_1_123; FJ517069	0.805	Freshwater	1

Oscillatoriales bacterium (0,0,0,0,1,0)	Cyanobacteria	uncultured cyanobacterium; F1BE01; FJ885984	0.827	Microbial mat (Y)	1
Oscillatoriales bacterium (0,0,0,0,1,0)	Cyanobacteria	uncultured cyanobacterium; DGGE gel band MB-06-MIC-B2; EU882217	0.908	Lake	1
Oscillatoriales bacterium (0,0,0,0,1,0)	Cyanobacteria	uncultured bacterium; A58; JQ027092	0.96	Carbon filter	1
Oscillatoriales bacterium (0,0,0,1,0,0)	Cyanobacteria	uncultured cyanobacterium; DGGE gel band MB-06-MIC-B2; EU882217	0.761	Lake	1
Oscillatoriales bacterium (0,0,1,0,0,0)	Cyanobacteria	uncultured cyanobacterium; LH-69; AB265892	0.813	Soil	1
Oscillatoriales bacterium (0,0,1,0,0,0)	Cyanobacteria	uncultured cyanobacterium; DGGE gel band MB-06-MIC-B13; EU882221	0.851	Lake	1
Oscillatoriales bacterium (0,0,1,0,0,0)	Cyanobacteria	uncultured Oscillatoriales cyanobacterium; UVmen2_33; JQ701187	1	Freshwater (M)	1
Oscillatoriales bacterium (0,1,0,0,0,0)	Cyanobacteria	uncultured cyanobacterium; DGGE gel band MB-06-MIC-B2; EU882217	0.853	Lake	1
Oscillatoriales bacterium (0,1,0,0,0,0)	Cyanobacteria	uncultured bacterium; BMB09; EF126218	0.944	Microbial mat	1
Oscillatoriales bacterium (1,0,0,0,0,0)	Cyanobacteria	uncultured cyanobacterium; DGGE gel band MB-06-MIC-B10; EU882215	0.745	Lake	1
Oscillatoriales bacterium (1,0,0,0,0,0)	Cyanobacteria	uncultured bacterium; Layer1_B08; HQ882868	0.937	Microbialite (CCB)	1
Prochlorothrix sp (0,0,0,1,0,0)	Cyanobacteria	uncultured Prochlorothrix sp.; Cont2_29; JQ701194	0.903	Freshwater (M)	1
Pseudanabaena sp (0,0,0,0,1,0)	Cyanobacteria	uncultured Pseudanabaena sp.; 40C_1_20; JQ701229	0.99	Freshwater (M)	1
Pseudanabaena sp (0,0,1,0,0,0)	Cyanobacteria	uncultured Pseudanabaena sp.; UVmas1_3; JQ701183	0.951	Freshwater (M)	1
Pseudanabaenaceae bacterium (0,0,0,0,1,0)	Cyanobacteria	uncultured Halomicronema sp.; Control_7; JQ701249	0.842	Freshwater (M)	1
Scytonemataceae bacterium (0,0,1,0,0,0)	Cyanobacteria	Scytonema sp. LCRSM-NDc; JN705691	0.949	Freshwater	1
Desulfovibrionales bacterium (0,0,0,0,0,1)	Deltaproteobacteria	uncultured delta proteobacterium; Alchichica_AQ1_1_1B_26; JN825525	0.926	Lake (A)	1
Desulfuromonadales bacterium (0,0,0,1,0,0)	Deltaproteobacteria	uncultured bacterium; CT1C2BE12; JQ427821	0.782	Soil	1
Desulfuromonadales bacterium (1,0,0,0,0,0)	Deltaproteobacteria	uncultured delta proteobacterium; A19YI04RM; FJ568484	0.826	Soil	1
Myxococcales bacterium (0,0,0,0,1,0)	Deltaproteobacteria	uncultured bacterium; V201-33; HQ114048	0.922	Biofilm	1
Myxococcales bacterium (0,1,0,0,0,0)	Deltaproteobacteria	uncultured Chondromyces sp.; 40-3_34; JQ700683	0.745	Freshwater (M)	1

Nannocystaceae bacterium (0,0,0,0,0,1)	Deltaproteobacteria	uncultured bacterium; F5K2Q4C04IOH5Y; GU912971	0.84	Sewage sludge	1
Nannocystaceae bacterium (0,0,0,0,0,1)	Deltaproteobacteria	uncultured bacterium; B0618R003_H08; AB659094	0.857	Soil	1
Syntrophobacterales bacterium (0,0,0,0,1,0)	Deltaproteobacteria	uncultured Desulfovibrio sp.; 40C_3_14; JQ701290	0.992	Freshwater (M)	1
Chromatiales bacterium (0,0,1,0,0,0)	Gammaproteobacteria	uncultured gamma proteobacterium; VERDEA80; FJ902650	0.783	Biofilm	1
Coxiellaceae bacterium (0,1,0,0,0,0)	Gammaproteobacteria	uncultured Coxiellaceae bacterium; UVmen1_5; JQ701274	0.994	Freshwater (M)	1
Legionellales bacterium (1,0,0,0,0,0)	Gammaproteobacteria	uncultured Coxiellaceae bacterium; UVmen1_46; JF413819	1	Freshwater (M)	1
Pseudomonas sp (0,0,0,1,0,0)	Gammaproteobacteria	uncultured eubacterium; GL178.11; AY038628	1	Sediment	1
Silanimonas sp (0,1,0,0,0,0)	Gammaproteobacteria	uncultured bacterium; NN07; JN869078	0.962	Lake	1
Sinobacteraceae bacterium (1,0,0,0,0,0)	Gammaproteobacteria	uncultured bacterium; CV109; DQ499329	0.754	Biofilm	1
Gemmatimonas sp (0,0,1,0,0,0)	Gemmatimonadetes	uncultured bacterium; SEAA1DB091; KC432048	0.893	Wetland	1
Gemmata sp (0,1,0,0,0,0)	Planctomycetes	uncultured bacterium; S2_125; JX406270	0.864	Lake	1
Phycisphaera sp (0,0,0,0,1,0)	Planctomycetes	uncultured bacterium; ELSC-TVG13-B68; GU220761	0.712	Hot Springs	1
Phycisphaera sp (0,1,0,0,0,0)	Planctomycetes	uncultured bacterium; Strom2G07; EU918120	0.775	Microbialite	1
Phycisphaera sp (0,1,0,0,0,0)	Planctomycetes	uncultured bacterium; SEAB1CH011; KC432190	0.897	Wetland	1
Phycisphaeraceae bacterium (0,0,0,0,1,0)	Planctomycetes	uncultured bacterium; S-73; KIST-JJY006; EF522844	0.701	Sewage sludge	1
Phycisphaeraceae bacterium (0,0,0,0,1,0)	Planctomycetes	uncultured Planctomycetales bacterium; Alchichica_AL31_2_1B_172; JN825612	0.736	Lake (A)	1
Phycisphaeraceae bacterium (0,0,1,0,0,0)	Planctomycetes	uncultured bacterium; N2_024; JX406211	0.941	Lake	1
Phycisphaeraceae bacterium (0,1,0,0,0,0)	Planctomycetes	uncultured bacterium; CyanoF10; EU917837	0.751	Microbialite	1
Phycisphaeraceae bacterium (0,1,0,0,0,0)	Planctomycetes	uncultured bacterium; CyanoF10; EU917837	0.803	Microbialite	1
Phycisphaeraceae bacterium (1,0,0,0,0,0)	Planctomycetes	uncultured bacterium; cpw1d616; HM921100	0.918	Groundwater	1
Phycisphaerales bacterium (0,0,1,0,0,0)	Planctomycetes	uncultured bacterium; 4-3; KC521963	0.786	Soil	1
Phycisphaerales bacterium (0,0,1,0,0,0)	Planctomycetes	uncultured planctomycete; HG-B01113; JN409100	0.834	Soil	1
Phycisphaerales bacterium (1,0,0,0,0,0)	Planctomycetes	uncultured planctomycete; OTU37/APA; 2F11; AM902605	0.624	Hot Springs	1
Phycisphaerales bacterium (1,0,0,0,0,0)	Planctomycetes	uncultured bacterium; ASNR-13; JQ809241	0.67	Sewage sludge	1
Planctomyces sp (0,0,0,0,0,1)	Planctomycetes	Planctomyces brasiliensis; LU4-23; JX307094	0.84	Groundwater	1

Planctomyces sp (0,0,0,0,1,0)	Planctomycetes	Planctomyces brasiliensis; LU4-23; JX307094	0.843	Oil well water	1
Planctomycetaceae bacterium (0,0,0,0,0,1)	Planctomycetes	uncultured Planctomyces sp.; Cont1_11; JQ701674	0.974	Freshwater (M)	1
Planctomycetaceae bacterium (0,0,0,0,1,0)	Planctomycetes	uncultured Planctomycetaceae bacterium; Flu2_6; JF413304	0.792	Freshwater (M)	1
Planctomycetaceae bacterium (0,0,0,1,0,0)	Planctomycetes	uncultured bacterium; 10NIIIH27; JN644181	0.906	Freshwater (M)	1
Planctomycetaceae bacterium (0,0,1,0,0,0)	Planctomycetes	uncultured bacterium; B139; JX967696	0.72	Soil	1
Planctomycetaceae bacterium (0,0,1,0,0,0)	Planctomycetes	uncultured planctomycete; as2-39; GU257592	0.923	Sewage sludge	1
Planctomycetaceae bacterium (0,1,0,0,0,0)	Planctomycetes	uncultured Planctomycetales bacterium; Alchichica_AL31_2_1B_172; JN825612	0.736	Lake	1
Planctomycetaceae bacterium (0,1,0,0,0,0)	Planctomycetes	uncultured bacterium; xyhpa1-58; HM051015	0.938	Lake	1
Planctomycetales bacterium (0,0,0,0,0,1)	Planctomycetes	uncultured bacterium; GJ16S2_G03; AB821100	0.698	Soil	1
Planctomycetales bacterium (0,0,1,0,0,0)	Planctomycetes	uncultured bacterium; AK4AB2_02G; GQ396923	0.772	Soil	1
Rhodopirellula sp (0,1,0,0,0,0)	Planctomycetes	uncultured bacterium; N2_024; JX406211	0.941	Lake	1
Singulisphaera sp (0,0,0,1,0,0)	Planctomycetes	uncultured planctomycete; P-B-An-25; JN867679	0.829	Wetland	1
Puniceicoccaceae bacterium (1,0,0,0,0,0)	Verrucomicrobia	uncultured organism; SBYC_4725; JN447017	0.589	Microbial mat (GN)	1
Subdivision3_genera_incertae_sedis (0,1,0,0,0,0)	Verrucomicrobia	uncultured bacterium; BJGMM-3s-171; JQ800931	0.768	Soil	1
Subdivision3_genera_incertae_sedis (0,1,0,0,0,0)	Verrucomicrobia	uncultured bacterium; FFCH15082; EU135450	0.881	Soil	1
Verrucomicrobiaceae bacterium (0,0,1,0,0,0)	Verrucomicrobia	uncultured Roseibacillus sp.; UVmen3_12; JQ701280	1	Freshwater (M)	1
