# Supplementary material for: Multivariate and Phylogenetic Analyses Assessing the Response of Bacterial Mat Communities from an Ancient Oligotrophic Aquatic Ecosystem to Different Scenarios of Long-Term Environmental Disturbance
Source: PLoS One. 2015 Mar 17;10(3):e0119741. doi: 10.1371/journal.pone.0119741 (PMC4363631; doi:10.1371/journal.pone.0119741)
Supplement: S2 Table — a-b) between the different environments (pools and treatments); c-d) only between the treatments. Tests are based on Bray-Curtis dissimilarity distances and 999 permutations. P (MC): P value based on Monte Carlo random draws. (PDF) [file pone.0119741.s007.pdf]

**Table S2. Results of Permutational multivariate analysis of variance (*adonis* function) and multivariate homogeneity of group dispersions analysis (*betadisper* function) of OTUs derived from 16S rRNA gene libraries data:** a-b) between the different environments (pools and treatments); c-d) only between the treatments. Tests are based on Bray-Curtis dissimilarity distances and 999 permutations. P (MC): P value based on Monte Carlo random draws.

a) Permutational multivariate analysis of variance (PERMANOVA) between the different environments (pools and treatments).

| Source | Df | Sums Sq | Mean Sq | F Model | R2 | P (MC) |
|---|---|---|---|---|---|---|
| Groups | 5 | 3.3121 | 0.66243 | 2.165 | 0.47425 | 0.001 *** |
| Residuals | 12 | 3.6718 | 0.30598 | | 0.52575 | |
| Total | 17 | 6.9839 | | | 1.00000 | |

b) Multivariate homogeneity of group dispersions analysis between the different environments (pools and treatments).

| Source | Df | Sums Sq | Mean Sq | F value | P (MC) |
|---|---|---|---|---|---|
| Groups | 5 | 0.017915 | 0.003583 | 0.4721 | 0.7902 |
| Residuals | 12 | 0.091071 | 0.0075893 | | |

c) Permutational multivariate analysis of variance (PERMANOVA) between the treatments.

| Source | Df | Sums Sq | Mean Sq | F Model | R2 | P (MC) |
|---|---|---|---|---|---|---|
| Groups | 4 | 2.6962 | 0.67405 | 2.1793 | 0.46573 | 0.001 *** |
| Residuals | 10 | 3.0930 | 0.30930 | | 0.53427 | |
| Total | 14 | 5.7892 | | | 1.00000 | |

d) Multivariate homogeneity of group dispersions analysis between the treatments.

| Source | Df | Sums Sq | Mean Sq | F value | P (MC) |
|---|---|---|---|---|---|
| Groups | 4 | 0.017170 | 0.0042924 | 0.7121 | 0.6122 |
| Residuals | 10 | 0.060275 | 0.0060275 | | |
